# Population structure, connectivity, and demographic history of an apex marine predator, the bull shark *Carcharhinus leucas*

**DOI:** 10.1002/ece3.5597

**Published:** 2019-09-30

**Authors:** Agathe Pirog, Virginie Ravigné, Michaël C. Fontaine, Adrien Rieux, Aude Gilabert, Geremy Cliff, Eric Clua, Ryan Daly, Michael R. Heithaus, Jeremy J. Kiszka, Philip Matich, John E. G. Nevill, Amy F. Smoothey, Andrew J. Temple, Per Berggren, Sébastien Jaquemet, Hélène Magalon

**Affiliations:** ^1^ UMR ENTROPIE (Université de La Réunion/IRD/CNRS) Université de La Réunion Saint Denis France; ^2^ UMR PVBMT CIRAD St Pierre France; ^3^ Laboratoire MIVEGEC (Université de Montpellier UMR CNRS 5290, IRD 229) Centre IRD de Montpellier Montpellier France; ^4^ Groningen Institute for Evolutionary Life Sciences (GELIFES) University of Groningen Groningen The Netherlands; ^5^ KwaZulu‐Natal Sharks Board Umhlanga Rocks South Africa; ^6^ School of Life Sciences University of KwaZulu‐Natal Durban South Africa; ^7^ EPHE CNRS UPVD USR 3278 CRIOBE PSL Research University Perpignan France; ^8^ Laboratoire d'Excellence CORAIL Perpignan France; ^9^ Oceanographic Research Institute Durban South Africa; ^10^ South African Institute for Aquatic Biodiversity Grahamstown South Africa; ^11^ Department of Biological Sciences Florida International University North Miami FL USA; ^12^ Environment Seychelles Victoria Seychelles; ^13^ NSW Department of Primary Industries Sydney Institute of Marine Science Mosman NSW Australia; ^14^ School of Natural and Environmental Sciences Newcastle University Newcastle‐upon‐Tyne UK

**Keywords:** ABC‐RF, microsatellite DNA, mitochondrial DNA, mito‐nuclear discordance, population genetics

## Abstract

Knowledge of population structure, connectivity, and effective population size remains limited for many marine apex predators, including the bull shark *Carcharhinus leucas*. This large‐bodied coastal shark is distributed worldwide in warm temperate and tropical waters, and uses estuaries and rivers as nurseries. As an apex predator, the bull shark likely plays a vital ecological role within marine food webs, but is at risk due to inshore habitat degradation and various fishing pressures. We investigated the bull shark's global population structure and demographic history by analyzing the genetic diversity of 370 individuals from 11 different locations using 25 microsatellite loci and three mitochondrial genes (*CR*, *nd4*, and *cytb*). Both types of markers revealed clustering between sharks from the Western Atlantic and those from the Western Pacific and the Western Indian Ocean, with no contemporary gene flow. Microsatellite data suggested low differentiation between the Western Indian Ocean and the Western Pacific, but substantial differentiation was found using mitochondrial DNA. Integrating information from both types of markers and using Bayesian computation with a random forest procedure (ABC‐RF), this discordance was found to be due to a complete lack of contemporary gene flow. High genetic connectivity was found both within the Western Indian Ocean and within the Western Pacific. In conclusion, these results suggest important structuring of bull shark populations globally with important gene flow occurring along coastlines, highlighting the need for management and conservation plans on regional scales rather than oceanic basin scale.

## Graphical abstract

We investigated the global population structure and demographic history by analyzing the genetic diversity of 370 individuals using 25 microsatellite loci and three mitochondrial genes (*CR*, *nd4*, and *cytb*). Both types of markers revealed clear clustering between sharks from the Western Atlantic and those from the Western Pacific and the Western Indian Ocean, with no contemporary gene flow. Between the Western Indian Ocean and the Western Pacific, microsatellite data suggested low differentiation while a substantial one was found with mitochondrial DNA. Integrating information from both types of markers and using Bayesian computation with a random forest procedure (ABC‐RF), this discordance was found to be due to either a complete lack of contemporary gene flow, or a complete lack of female gene flow along with some contemporary male effective dispersal.

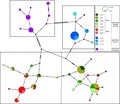

## 1 INTRODUCTION

Delineating populations and their connectivity by gene flow (i.e., effective dispersal) is of primary importance for the conservation and management of endangered and/or exploited species (Begg, Friedland, & Pearce, 1999; Moritz, 1994; Palsbøll, Bérubé, & Allendorf, 2007). In marine species, genetic analyses allow for stocks to be defined, species exploitation status to be assessed, and genetic diversity underlying recruitment potential and species adaptability to be preserved (Begg et al., 1999; Hilborn, Quinn, Schindler, & Rogers, 2003; Palumbi, 2003). Once genetically distinct groups (i.e., populations) that may be managed independently are identified, estimating abundance and the number of individuals effectively exchanged among populations is needed to assess population viability and resilience (Frankham, 2010; Schwartz, Luikart, & Waples, 2007). Among highly mobile, wide‐ranging species, such as marine megafauna (e.g., marine mammals, seabirds, turtles, sharks, and rays) and large‐bodied teleosts, such studies are particularly important because of exposure to anthropogenic pressures (Halpern et al., 2008; Payne, Bush, Heim, Knope, & McCauley, 2016) and the key roles many play within food webs (Bowen, 1997; Estes, 1979; Heithaus, Frid, Wirsing, & Worm, 2008; Katona & Whitehead, 1988).

Studies of population structure and connectivity are challenging because commonly used direct approaches (mark–recapture, satellite, and acoustic tracking) are often difficult to use for pelagic marine species. This difficulty leads to small sample sizes (Grothues, 2009) and an underestimation of individual movements (Ng, Able, & Grothues, 2007; Thorsteinsson, 2002). Therefore, indirect methods based on the conceptual framework of population genetics have been increasingly used to address ecological and evolutionary questions in such species. First, genetic methods allow the assessment of population structure resulting from evolutionary forces shaping allele frequencies within and among populations (mutation, genetic drift, migration, and selection; Wright, 1931). At neutral loci, while gene flow homogenizes allele frequencies and limits population differentiation, genetic drift promotes population differentiation by randomly fixing alleles (Hartl & Clark, 1997). Second, genetic methods can provide estimates of the effective population size (*N*_e_; Wright, 1931). This parameter represents the size of an idealized Wright–Fisher population affected by genetic drift at the same rate per generation found in the population of interest. Combined with the mutation rate (*µ*), *N*_e_ provides an estimate of population genetic diversity (4*N*_e_ *µ* for the diploid autosomal part of the genome and *N*_e_ *µ* for the haploid mitochondrial genome). *N*_e_ is also related to the number of breeders per generation (Waples, Antao, & Luikart, 2014) and has been shown to correlate with a population's ability to adapt to environmental changes (Hare et al., 2011). *N*_e_ has thus been increasingly used in conservation and management to estimate the health status of a population and its ability to recover when depleted (Frankham, Briscoe, & Ballou, 2010).

Marine species characterized by large populations commonly show weak genetic structuring at neutral loci. In large populations, even a low dispersal rate can lead to weak population genetic structure because the number of migrants is not negligible. Also, genetic drift is limited in these species due to their large population sizes (Bailleul et al., 2018; Gagnaire et al., 2015; Palumbi, 1992). Weak genetic structuring may result from the existence of large isolated populations or, conversely, the existence of one large panmictic population. Identifying which situation is driving population structure can be challenging, but recent developments are providing the necessary analytical resolution. Incorporation of migration into simulation models, combined with new approximate Bayesian computation algorithm relying on random forest (i.e., ABC‐RF), allows comparisons and selection of alternative demographic models that best fit the observed dataset (Pudlo et al., 2016; Raynal et al., 2017). ABC‐RF provides estimates of the posterior probability of the selected model and the parameters of interests, such as migration rates between populations and effective population size (Pudlo et al., 2016; Raynal et al., 2017). For both model choice and parameter estimates, ABC‐RF is more accurate and requires a smaller number of simulated datasets than previous ABC methods (Fraimout et al., 2017; Pudlo et al., 2016; Raynal et al., 2017).

Many large sharks face considerable exploitation, and populations have declined globally in recent decades (Dulvy et al., 2014). The bull shark *Carcharhinus leucas* is caught in recreational, subsistence, and targeted commercial fisheries, as well as bycatch throughout its range (Aguilar et al., 2014; Almeida, McGrath, & Ruffino, 2001; Bonfil, 1997; Branstetter & Stiles, 1987; Clarke, Magnussen, Abercrombie, McAllister, & Shivji, 2006; Doukakis et al., 2010; Temple et al., 2018). In several locations, the bull shark has also been the subject of lethal risk reduction programs due to attacks on humans (Cliff & Dudley, 1991; Dudley, 1997; Dudley & Simpfendorfer, 2006; Lagabrielle et al., 2018). This high‐trophic level predator inhabits warm temperate and tropical waters worldwide, and plays an important role in coastal and estuarine ecosystems (Daly, Froneman, & Smale, 2013; Matich, Heithaus, & Layman, 2011; Trystram, Rogers, Soria, & Jaquemet, 2017). Therefore, stock assessments and evaluation of genetic structure is a priority step for this species.

Population structuring and connectivity in large sharks vary in relation to environmental features, movement ecology, and habitat preferences (Dudgeon et al., 2012; Heist, 2005). Oceanic species generally exhibit high levels of genetic connectivity, including across ocean basins (e.g., basking shark *Cetorhinus maximus*; Hoelzel, Shivji, Magnussen, & Francis, 2006), while coastal species tend to exhibit more structure (e.g., blacktip reef shark *Carcharhinus melanopterus* [Mourier & Planes, 2013; Vignaud et al., 2014] and scalloped hammerhead shark *Sphyrna lewini* [Duncan, Martin, Bowen, & De Couet, 2006]). Despite the bull shark being able to undergo long‐distance migrations (Brunnschweiler, Queiroz, & Sims, 2010; Daly, Smale, Cowley, & Froneman, 2014; Heupel et al., 2015; Kohler & Turner, 2001; Lea, Humphries, Clarke, & Sims, 2015), its dispersal may be restricted, as is suggested by high genetic differentiation observed between Fiji, the Atlantic, and Indo‐West Pacific Oceans (Testerman, 2014). However, no genetic subdivision has been identified among populations within a continental basin (Karl, Castro, Lopez, Charvet, & Burgess, 2011; Testerman, 2014; Tillett, Meekan, Field, Thorburn, & Ovenden, 2012). This low connectivity has been suggested to result from (a) oceanic waters acting as a barrier and (b) possible female philopatry to natal nurseries.

Many sharks exhibit philopatry, returning either to specific feeding areas (e.g., the tiger shark *Galeocerdo cuvier*; Meyer, Clark, Papastamatiou, Whitney, & Holland, 2009; Meyer, Papastamatiou, & Holland, 2010) or nursery grounds (Hueter, Heupel, Heist, & Keeney, 2005; Portnoy & Heist, 2012; Speed, Field, Meekan, & Bradshaw, 2010). These behaviors may be sex‐specific and, for many coastal sharks, result in population structure at smaller geographic scales than would be expected based on locomotive abilities (Chapman, Feldheim, Papastamatiou, & Hueter, 2015). Bull sharks use estuaries and rivers for nurseries (Heupel, Yeiser, Collins, Ortega, & Simpfendorfer, 2010; Ortega, Heupel, Van Beynen, & Motta, 2009; Snelson, Mulligan, & Williams, 1984), making female philopatry likely throughout their range (Karl et al., 2011; Tillett et al., 2012).

Estimates of long‐term effective population size of bull sharks vary among studies and locations, but are likely in the order of 100,000 individuals (Karl et al., 2011; Testerman, 2014), which represents potentially greater genetic diversity than other shark species, for which estimates of *N*_e_ are in the order of 10,000–50,000 individuals (e.g., basking sharks, Hoelzel et al., 2006; sicklefin lemon shark, Schultz et al., 2008). This may suggest that (a) bull shark populations are not severely depleted and/or (b) that fishery pressures are too recent to be detected through genetic analyses (Karl et al., 2011; Testerman, 2014).

**Figure 1:**
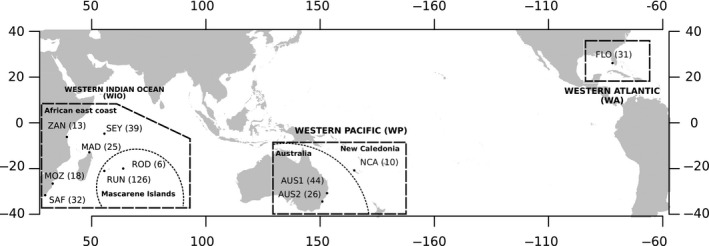
Map of bull shark (*Carcharhinus leucas*) sampling locations (ZAN, Zanzibar; SEY, Seychelles; MOZ, Mozambique; SAF, South Africa; MAD, Madagascar; RUN, Reunion Island; ROD, Rodrigues Island; AUS1, Clarence River, Australia; AUS2, Sydney Harbour, Australia; NCA, New Caledonia; FLO, Florida). Sample sizes are in brackets. Boxes indicate ocean basins and dotted lines delineate regions

To date, few studies have investigated bull shark genetic structure and have relied either on (a) extensive sampling on a global scale using only nuclear markers (Testerman, 2014), or (b) a locally intensive sampling (either restricted to the Atlantic or Northern Australia), using relatively few nuclear and mitochondrial markers (3–5 microsatellites along with 1 or 2 mitochondrial genes; Karl et al., 2011; Tillett et al., 2012). Thus, improving our understanding of bull shark population structuring and connectivity across ocean basins is needed. Combining the information from two types of molecular markers (25 microsatellite loci and three mitochondrial genes [*CR*, *nd4*, and *cytb*]), we analyzed the genetic variation in 370 bull sharks from 11 locations in the Western Indian Ocean, the Western Pacific, and the Western Atlantic, including both continental coasts and oceanic islands (Figure 1). By including new locations and increasing the number of markers presenting different modes of evolution, our objective was to combine classical population genetic analyses with coalescent‐based approximate Bayesian computation approaches (Beaumont, 2010; Csilléry, Blum, Gaggiotti, & François, 2010). This was performed to delineate bull shark populations and assess their demographic history and connectivity, using model selection analyses to refine the evolutionary history of this species. Specifically, the objectives were to:

1. Expand our understanding of the genetic structure previously documented by Testerman (2014) in order to delineate genetic clusters at different scales (e.g., within vs. among ocean basins) that should be managed separately;
2. Decipher whether contemporary migration occurs among defined clusters; and
3. Estimate the effective population sizes of these clusters.

## 2 MATERIALS AND METHODS

### 2.1 Sampling

Tissue samples were collected in the Western Indian Ocean (WIO), the Western Pacific (WP), and the Western Atlantic (WA; Figure 1). In the WIO, samples came from continental coasts and oceanic islands: Zanzibar (ZAN), *n* = 13; Mozambique (MOZ), *n* = 18; South Africa (SAF), *n* = 32; the Seychelles (SEY), *n* = 39; Madagascar (MAD), *n* = 25; Reunion Island (RUN), *n* = 126; and Rodrigues Island (ROD), *n* = 6. Samples from the Western Pacific were collected in two regions along the east coast of Australia [Clarence River (AUS1; *n* = 44) and Sydney Harbour (AUS2; *n* = 26), New South Wales] and in New Caledonia (NCA, *n* = 10). Most of the samples came from biopsies made on individuals caught in the wild for commercial, risk reduction, or scientific purposes. Samples from Madagascar came from carcharhinid jaws or teeth found in markets and a posteriori confirmed as belonging to bull sharks by sequencing the mtDNA control region (*CR*). Finally, in the Western Atlantic, samples were collected in the Shark River estuary in the Florida Coastal Everglades (Florida, US; FLO; *n* = 31). All samples were collected on subadult or adult individual, except in Florida where they were young‐of‐the‐year and juveniles. In total, 370 samples were collected and preserved in 90% ethanol until laboratory analyses (Figure 1).

### 2.2 Laboratory procedures

Genomic DNA was extracted using Qiagen DNeasy Blood & Tissue Kit (Qiagen, Hilden, Germany) following manufacturer instructions.

Each sample was genotyped at 25 microsatellite loci. Twenty loci were species‐specific (Cl01 to Cl20; Pirog, Blaison, Jaquemet, Soria, & Magalon, 2015) and were analyzed following the procedure described in Pirog et al. (2015). The remaining five microsatellite loci were originally developed for the tiger shark *G. cuvier* [Gc01 (Pirog, Jaquemet, Blaison, Soria, & Magalon, 2016); TIG10 (Mendes et al., 2016)], the sandbar shark *Carcharhinus plumbeus* (Cpl166; Portnoy, Mcdowell, Thompson, Musick, & Graves, 2006), the Australian blacktip shark *Carcharhinus tilsoni* (Ct05; Ovenden, Street, & Broderick, 2006), and the lemon shark *Negaprion brevirostris* (Ls24; Feldheim, Gruber, & Ashley, 2001) and successfully cross‐amplified in the bull shark. These loci were indirectly labeled using 6‐FAM, PET, VIC, or NED fluorochromes, and PCRs were carried out following Gélin, Postaire, Fauvelot, and Magalon (2017). The 25 loci were multiplexed post‐PCR in five panels (Appendix S1). The allelic sizes of the PCR products were separated on an ABI 3730XL capillary sequencer at the Plateforme Gentyane (INRA) and scored with GeneMapper v.4.0 (Applied Biosystems) using the Genescan LIZ‐500 size standard (Applied Biosystems). Some samples were analyzed twice to check the consistency of the results.

The mtDNA control region (*CR*) was PCR‐amplified using the set of primers GWF (Pardini et al., 2001) and CL2 (Tillett et al., 2012), the nicotinamide adenine dinucleotide dehydrogenase (NADH) subunit 4 (*nd4*) using primers nd4 (Arevalo, Davis, & Sites, 1994) and H12293_LEU (Inoue, Miya, Tsukamoto, & Nishida, 2001), and the cytochrome b (*cytb*) with primers GluDG and C61121H (Naylor, Ryburn, Fedrigo, & Lopez, 2005). This was performed for subsets of the sampling: 266 individuals for *CR*, 255 individuals for *nd4*, and 227 for *cytb*.

PCRs were performed in a total volume of 25 µl: 1× of MasterMix (Applied Biosystems), 0.3 µM of forward and reverse primers, and 1.6 ng/µl of genomic DNA. The thermocycling program for *CR* contained an initial denaturing step at 94°C for 5 min, 35 cycles × (94°C for 30 s, 56°C for 30 s, 72°C for 1 min 30 s), and a final extension step at 72°C for 5 min. For *cytb*, the same program was used, except that the annealing temperature was set to 53°C. For *nd4*, the annealing temperature was 50°C and the elongation step was 45 s. Amplicons were sequenced directly with primers used for PCR on a capillary sequencer ABI 3730XL (Applied Biosystems) by Genoscreen.

### 2.3 Genetic diversity analysis

Among the individuals from Madagascar, 12 out of 25 samples were kept for data analyses, because the remaining samples extracted from teeth exhibited high amounts of missing data (more than 50%) due to low‐quality DNA.

Null alleles were assessed with microchecker v.2.2.3 (Van Oosterhout, Hutchinson, Wills, & Shipley, 2004). Linkage disequilibrium (LD) between pairs of loci was tested using a likelihood‐ratio test with 10,000 permutations in arlequin v.3.5.1.2 (Excoffier & Lischer, 2010). Diversity indices such as the number of alleles per locus *N*_a_, observed and expected heterozygosities (*H*_O_ and *H*_E_), and inbreeding coefficient *F*_IS_ (Weir & Cockerham, 1984) were estimated using fstat v.2.9.3.2 (Goudet, 1995). Departure from Hardy–Weinberg equilibrium (HWE) at each microsatellite locus was tested using 5,000 permutations in fstat v.2.9.3.2 (Goudet, 1995). The mean allelic richness *A*_r_ and the mean private allelic richness *A*_rp_ were calculated using a rarefaction method, as implemented in hp‐rare v.1.0 (Kalinowski, 2005). This method accounts for differences in sample size by standardizing *A*_r_ and *A*_rp_ values across sampled locations by resampling the lowest number of genotypes available (i.e., 12 haploid gene copies or six diploid genotypes in Rodrigues Island) in each location.

Mitochondrial sequences were checked and aligned using Geneious v.8.1.2 (Kearse et al., 2012). Alignments were performed using the MAFFT method (Katoh, Misawa, Kuma, & Miyata, 2002) for each marker separately first and then for the concatenated sequence (*CR‐nd4‐cytb*). Diversity indices (i.e., number of haplotypes, number of segregating sites, haplotype (*h*), and nucleotide (*π*) diversities) were calculated for the concatenated alignment and for each marker separately using dnasp v.5.10.1 (Librado & Rozas, 2009).

Detection of partitioning schemes within the concatenated sequence *CR‐nd4‐cytb* and of substitution models was performed using partitionfinder v.2.1.1 (Guindon et al., 2010; Lanfear, Frandsen, Wright, Senfeld, & Calcott, 2017). We used beast v.1.8.4 (Drummond, Suchard, Xie, & Rambaut, 2012) to reconstruct phylogenetic relationships and infer divergence times on the mitochondrial concatenated sequence *CR‐nd4‐cytb*. Bayesian Markov chain Monte Carlo (MCMC) analyses were performed assuming a HKY85 + I model of substitution as the latter was shown to best fit the data. The rate of variation among sites was modeled with a discrete gamma distribution with four rate categories. We assumed an uncorrelated lognormal relaxed clock to account for rate variation among lineages. To minimize prior assumptions about demographic history, we adopted an extended Bayesian skyline plot (EBSP) approach in order to integrate data over different demographic histories. Trees were calibrated using two methods. First, an analysis was performed adding a sequence of *S. lewini* (mitochondrion available in GenBank; accession number JX827259), and the tree was calibrated using the divergence date between *Carcharhinus* and *Sphyrna* genera, 38 millions years ago (Mya), estimated from fossil data (Maisey, 1984). Second, the tree was calibrated using the closure of the Isthmus of Panama as the divergence time of bull shark populations from the Western Atlantic and the Indo‐Pacific, 3.1–3.5 Mya (Coates, Collins, Aubry, & Berggren, 2004; Coates et al., 1992). For each analysis, a normal prior distribution was set for the calibrated node (mean ± *SD*: 38 ± 7 and 3.5 ± 0.4, respectively). Evolutionary model parameters were then estimated, with samples drawn from the posterior every 10^5^ MCMC steps over a total of 10^8^ steps from five independent runs. The first 10^7^ steps were discarded as burn‐in. Good mixing and convergence were assessed using tracer v.1.6 (Rambaut, Suchard, Xie, & Drummond, 2014), and the best tree was selected using the maximum clade credibility option with treeannotator v.1.8.4 (Drummond et al., 2012) and viewed with figtree v.1.4.0 (http://tree.bio.ed.ac.uk/software/figtree/). To further evaluate phylogenetic relationships among haplotypes, a TCS statistical parsimony network (Clement, Posada, & Crandall, 2000) was constructed using popart v.1.7 (Leigh & Bryant, 2015).

### 2.4 Population genetic structure

Two complementary clustering methods were used to investigate population structure in the bull shark. First, Bayesian clustering analyses were performed using structure v.2.3.4 (Falush, Stephens, & Pritchard, 2003; Pritchard, Stephens, & Donnelly, 2000). For any given number of clusters (*K*) between 1 and 10, individual assignment probabilities to each cluster were determined so as to minimize departures from HWE within clusters and maximize LD between them. Two analyses were performed, with and without the LOCPRIOR model, which uses prior sampling location information in the Bayesian clustering to detect genetic population structure that might be weaker (Hubisz, Falush, Stephens, & Pritchard, 2009). Conditions were set to 10^6^ chain length after a burn‐in of 5 × 10^5^, and 10 chains were run for each *K* assuming correlated allele frequencies and the admixture model. For a given *K*, distinct modes were identified, and for each mode and each individual, the assignment probabilities to each cluster were averaged using Clumpak (Kopelman, Mayzel, Jakobsson, Rosenberg, & Mayrose, 2015). Second, a discriminant analysis of principal components (DAPC Jombart, Devillard, & Balloux, 2010), which does not rely on HWE or LD contrary to structure, was performed to check whether similar clustering patterns were identified. This method summarizes the genetic variation of the microsatellite allele frequencies using a principal component analysis as a prior step to a discriminant analysis and defines clusters such as to minimize variations within them and maximize differentiation between them. DAPC was applied using the *adegenet* package (Jombart, 2008) for R (R Core Team, 2017). Methods traditionally used to detect the most likely number of clusters according to the analysis performed (Structure and DAPC) might provide different outputs for the same dataset. To cope with these inconsistencies, we chose to consider the highest number of clusters and the individual assignments that were retrieved by both analyses. Moreover, in a hierarchical approach, these analyses were repeated on each cluster found separately. Commonly, using Structure and DAPC, when the finest level of structuring is reached, adding a supplementary cluster leads to inconclusive assignments with individuals assigned to several clusters in the same proportions.

Analyses of molecular variance (AMOVAs; Cockerham, 1969, 1973) were performed to estimate the genetic variation due to the partitioning in clusters (identified with the TCS haplotype network for the mitochondrial data and with structure and DAPC for microsatellite data), the variation within clusters among sampling locations, and the variation within sampling locations. AMOVAs were performed with arlequin v.3.5.1.2 (Excoffier & Lischer, 2010), and significance of fixation indices was tested using a nonparametric approach with 10,000 permutations (Excoffier, Smouse, & Quattro, 1992).

To assess population differentiation between pairs of sampling locations, *F*_ST_ (Weir & Cockerham, 1984) and *D*_est_ (Jost, 2008) were estimated for the microsatellites using arlequin v.3.5.1.2 (Excoffier & Lischer, 2010) and *DEMEtics* v.0.8–7 (Gerlach, Jueterbock, Kraemer, Deppermann, & Harmand, 2010), respectively. The *D*_est_ is based on the effective number of alleles and is less affected by within‐population variation compared with *F*_ST_. For the mitochondrial dataset, the Φ_ST_ (Slatkin, 1995) was estimated using arlequin v.3.5.1.2 (Excoffier & Lischer, 2010). Significance of pairwise population differentiation indices was tested using 10,000 permutations.

### 2.5 Demographic history and variations of effective population sizes

#### 2.5.1 Neutrality tests

To test for departures from a constant population size (Ramos‐Onsins & Rozas, 2000), the summary statistics Tajima's *D* (Tajima, 1989) and Fu's *F*_S_ (Fu, 1997) were estimated from the concatenated mitochondrial dataset with Arlequin v.3.5.1.2 (Excoffier & Lischer, 2010), with significance tested implementing 10^5^ simulated samples.

#### 2.5.2 ABC‐RF analyses

##### Demographic scenarios

**Figure 2:**
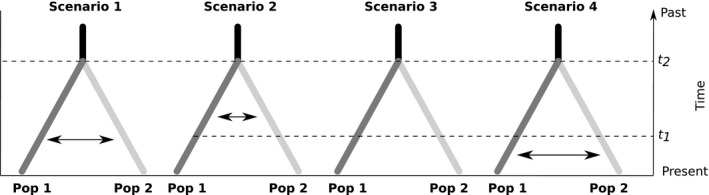
Graphical representations of the four scenarios depicting possible divergence histories for each pair of bull shark populations: FLO‐RUN, FLO‐AUS1, and RUN‐AUS1. The time was measured backward in generations before present. In black, is represented the ancestral population of effective population size *N*_anc_; in dark gray, population 1 of effective population size *N*_1_ and in light gray, population 2 of effective population size *N*_2_. Double arrows represent bidirectional migration events. *t*_2_, time of divergence; *t*_1_, start and end of the isolation period for Scenario 2 and Scenario 4, respectively

Combining the information given by both types of markers (microsatellites and mtDNA), we attempted to infer the intensity of gene flow between the Western Indian Ocean and the Western Pacific populations and the effective sizes of the delineated populations. To do so, historical scenarios of population divergence differing in the assumptions regarding migration were compared in a Bayesian framework using random forests to identify the best model of population split and to estimate the model parameters (ABC‐RF; Pudlo et al., 2016; Raynal et al., 2017). The Western Atlantic population deviated from a panmictic population, which might bias the analysis. It was therefore not included in the ABC‐RF analysis. Pooling individuals from different sampling locations, even with nonsignificant pairwise differentiation values, may bias results (Lombaert et al., 2014). Hence, the two regions were represented by the sampling location with the highest number of individuals, that is Reunion Island (RUN) for WIO and Eastern Australia (Clarence River, AUS1) for WP. Four demographic scenarios were built (Figure 2), all of them starting with an ancestral population from which both observed populations diverged. Scenarios then differed as to the occurrence of migration during divergence. Scenario 1 assumed constant recurrent migration from the split to present. In Scenario 2, the split was followed by a period of recurrent migration, itself followed by a period of isolation. In Scenario 3, populations diverged in isolation. Finally, Scenario 4 assumed that populations first went through a period of isolation before engaging in a period with recurrent migration. In all scenarios, recurrent migration was bidirectional but not necessarily symmetric.

##### Model choice

For each scenario, we simulated 200,000 microsatellite and mitochondrial datasets using Fastsimcoal (Laval & Excoffier, 2004). To account for both types of markers having different sample sizes, we applied a two‐step procedure (bash scripts available upon request). Microsatellite datasets were first simulated with parameters drawn in the prior distributions described in Appendix [Link], [Link] (Tables S2.1 and S2.2). The mitochondrial datasets were subsequently simulated using the same historical parameters (divergence times, starting time, and ending time of the migration period) as for microsatellites, but with different sample sizes and, importantly, different demographic and genetic parameters (effective sizes, migration rates, and mutation rates). We thus estimated different migration rates and effective sizes for microsatellite and mtDNA. Because of the lack of knowledge on effective sizes and historical divergence of bull shark populations, broad parameter ranges were chosen. Simulated datasets were described using 19 summary statistics (Appendix [Link], [Link]) related to the genetic polymorphism of both types of loci using Arlsumstat (Excoffier & Lischer, 2010). For both markers, we computed the mean number of alleles over loci *K* and the mean of Nei's gene diversity *H* for each population and the pairwise *F*_ST_ between populations. For microsatellite markers only, the mean over loci of the modified Garza–Williamson index *MGW* were computed for each population and the mean delta mu‐square *δµ* ^2^ (square difference in mean microsatellite allele length between pairs of populations) between the sampled populations. For mitochondrial markers only, the mean number of pairwise differences *π*, the Tajima's *D*, and the Fu's *Fs* were computed for each population. Prior checking was performed using principal components analyses (PCAs) to project the summary statistics obtained from the simulated and the observed datasets, and confirming the observed value of each statistic falls well within the distribution of the simulated datasets. The scenarios that best fitted the data were identified using the random forest procedure implemented in the *abcrf* R package (Marin, Raynal, Pudlo, Robert, & Estoup, 2017) using 20,000 of the simulated datasets, with the analysis replicated 10 times. The linear discriminant analysis (LDA) axes were added to the 19 summary statistics mentioned earlier to summarize the datasets, as it has been shown to improve the discrimination between scenarios (Pudlo et al., 2016). The best scenario was identified by analyzing the posterior probabilities of each scenario over the replicate analyses (Fraimout et al., 2017). The prior error rates of the best scenario (i.e., the probability of choosing a wrong model when drawing model index and parameter values into priors; Pudlo et al., 2016) were averaged over the replicate analyses (Fraimout et al., 2017).

##### Parameter estimations

Parameter values were subsequently inferred using ABC random forests as developed by Raynal et al. (2017), using 100,000 datasets simulated under the best scenario. To test the performance of the method in estimating parameters, we used 1,000 pseudo‐observed datasets on which the estimation procedure was applied to measure the precision of the estimation procedure. From these values, the 95% confidence interval (CI) and the normalized mean square error NMSE were computed. Parameter inference analyses were replicated two times to ensure consistency of ABC‐RF analyses.

## 3 RESULTS

### 3.1 Genetic diversity analysis

Null alleles were detected for several loci in several sampling locations, but were not constant among locations and were not correlated with significant deviations from HWE. All loci were thus kept for further analyses. For microsatellite loci, a globally significant LD was detected for only four of 3,300 tests after FDR correction (0.12%, *p* < .05), and consequently, all loci were considered independent. The average number of alleles (±*SE*) per location ranged from 2.88 ± 0.45 in New Caledonia and 2.88 ± 0.66 in Rodrigues Island to 4.56 ± 0.19 in Reunion Island. Mean allelic richness corrected by a standardized sample size of 6 diploid individuals remained relatively constant among sampling locations, varying from 2.56 ± 0.34 in Florida to 2.88 ± 0.66 in Rodrigues Island. *H*_E_ and *H*_O_ varied from 0.42 ± 0.05 in Australia (AUS2) to 0.54 ± 0.09 in Rodrigues Island and from 0.37 ± 0.05 in Florida to 0.56 ± 0.10 in Rodrigues Island, respectively (Table 1). Significant deviation from HWE was observed only for Florida (*F*_IS_ = 0.17, *p* < .01), which could be linked to sampling within a single nursery (sampling of juveniles within a same nursery, which could be related). The mean private allelic richness varied from 0.01 ± 0.01 in Zanzibar to 0.15 ± 0.13 in Rodrigues Island in the WIO and the WP, and was of 0.67 ± 0.29 in Florida (Table 1).

**Table 1:** Summary statistics for each bull shark sampling location averaged across 25 microsatellite loci or for the 2,516 bp concatenated mitochondrial sequence *CR‐nd4‐cytb*

	ZAN	SEY	MOZ	SAF	MAD	RUN	ROD	AUS1	AUS2	NCA	FLO
Microsatellite											
*N*	13	39	18	32	12	126	6	44	26	10	31
*N*_a_	3.12 (0.48)	4.08 (0.38)	3.52 (0.39)	3.84 (0.34)	3.44 (0.48)	4.56 (0.19)	2.88 (0.66)	4.04 (0.38)	3.76 (0.38)	2.88 (0.45)	3.88 (0.83)
*A*_r_	2.63 (0.33)	2.75 (0.18)	2.75 (0.29)	2.77 (0.21)	2.82 (0.35)	2.78 (0.10)	2.88 (0.66)	2.77 (0.19)	2.70 (0.24)	2.58 (0.39)	2.56 (0.34)
*A*_rp_	0.01 (0.01)	0.07 (0.02)	0.03 (0.02)	0.07 (0.01)	0.06 (0.04)	0.05 (0.00)	0.15 (0.13)	0.06 (0.01)	0.06 (0.02)	0.03 (0.03)	0.67 (0.29)
*H*_O_	0.45 (0.06)	0.44 (0.03)	0.44 (0.06)	0.43 (0.04)	0.46 (0.07)	0.42 (0.02)	0.56 (0.10)	0.47 (0.03)	0.39 (0.05)	0.47 (0.08)	0.37 (0.05)
*H*_E_	0.48 (0.06)	0.45 (0.03)	0.43 (0.06)	0.45 (0.04)	0.44 (0.06)	0.44 (0.02)	0.54 (0.09)	0.47 (0.03)	0.42 (0.05)	0.47 (0.07)	0.44 (0.05)
*F*_IS_	0.06	0.02	−0.02	0.03	−0.03	0.03	−0.04	0.00	0.06	−0.01	0.17**
*CR‐nd4‐cytb*											
*N_s_*	13	36	18	25	8	38	6	23	14	7	30
*H*	4	9	10	8	6	12	2	3	2	2	8
*h*	0.69 (0.03)	0.74 (0.01)	0.88 (0.01)	0.81 (0.01)	0.93 (0.03)	0.81 (0.01)	0.33 (0.09)	0.17 (0.02)	0.49 (0.02)	0.48 (0.06)	0.80 (0.01)
*S*	8	12	13	12	10	17	1	6	3	5	13
*π*	0.00143 (0.00024)	0.00133 (0.00013)	0.00154 (0.00021)	0.00159 (0.00018)	0.00212 (0.00046)	0.0015 (0.00014)	0.00013 (0.00007)	0.00024 (0.00005)	0.00059 (0.00011)	0.00095 (0.00025)	0.00131 (0.00014)

Note

In brackets are indicated standard error values. ZAN, Zanzibar; SEY, Seychelles; MOZ, Mozambique; SAF, South Africa; MAD, Madagascar; RUN, Reunion Island; ROD, Rodrigues Island; AUS1, Clarence River, Australia; AUS2, Sydney Harbour, Australia; NCA, New Caledonia; FLO, Florida.

*N,* number of individuals genotyped with less than 50% missing data; *N*_a_, number of alleles averaged across loci; *A*_r_, mean rarified allelic richness based on a standardized sample size of six diploid individuals (ROD); *A*_rp_, mean rarified private allelic richness based on a standardized sample size of six diploid individuals (ROD); *H*_O_, mean observed heterozygosity; *H*_E_, mean expected heterozygosity; *F*_IS_, inbreeding coefficient and significant deviations from Hardy–Weinberg equilibrium (**p < .01); *N*_s_, number of individuals sequenced; *H*, number of haplotypes; *h*, haplotype diversity; *S*, number of polymorphic sites; *π*, nucleotide diversity.

Summary statistics for each mitochondrial gene are presented in Appendix S3 (GenBank Accession numbers MN227021–MN227067). We obtained sequences of 923 bp for *CR*, 672 pb for *nd4*, and 921 bp for *cytb* and resolved 19, 13, and 17 haplotypes with 18, 22, and 23 polymorphic sites, respectively. Total haplotype diversities (*h*) were of the same order for each gene, varying from 0.80 ± 0.00 for *CR* and *cytb* to 0.86 ± 0.00 for *nd4*. Total nucleotide diversity (*π*) was higher for *nd4* (0.00834 ± 0.00003) than for *CR* and *cytb* (0.00448 ± 0.00001 and 0.00426 ± 0.00002, respectively).

**Figure 3:**
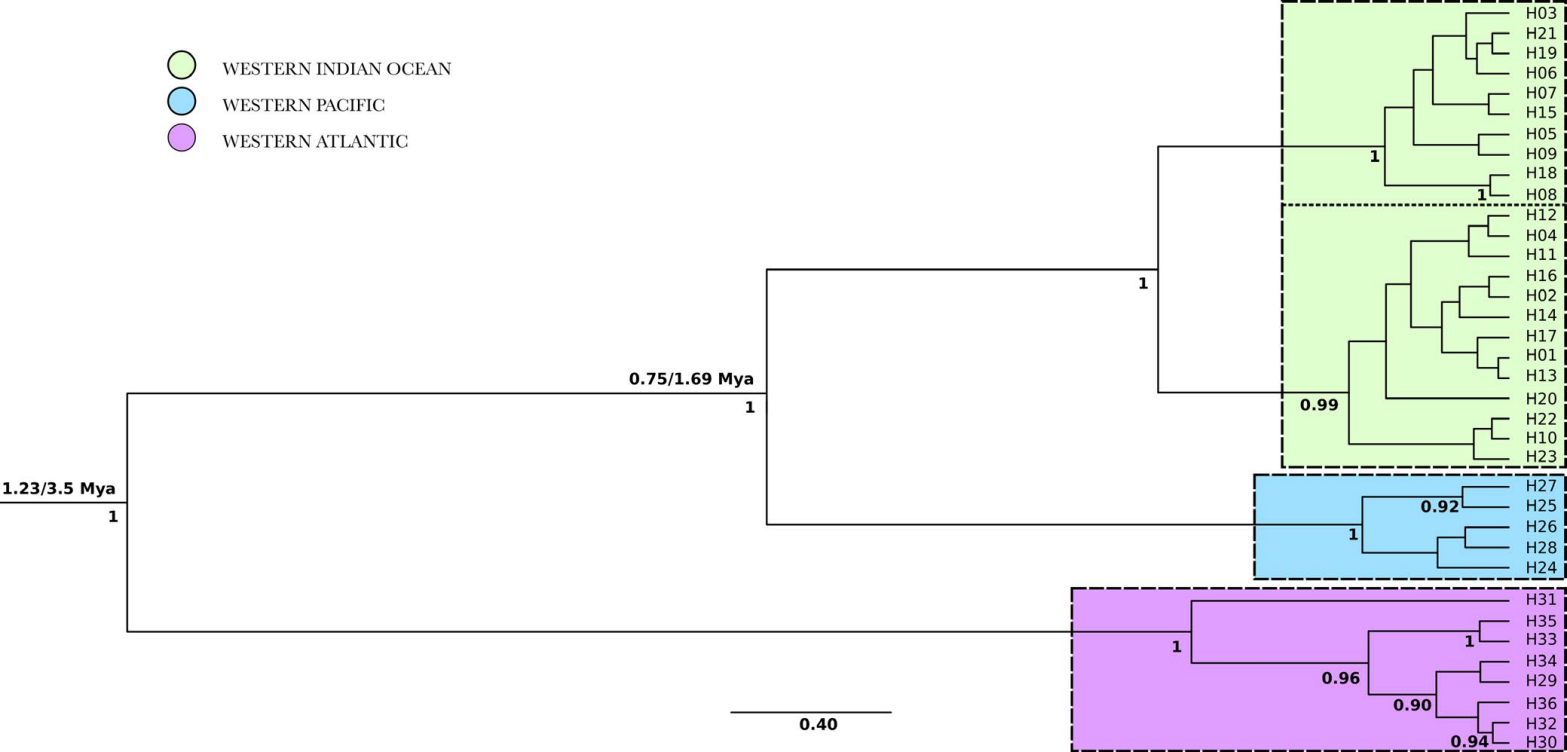
Maximum clade credibility tree of the mitochondrial concatenated sequence *CR‐nd4‐cytb* for the bull shark. Only the different haplotypes are represented. Boxes delineate lineages discussed in the text. Below branches, are indicated node supports above 0.90; above branches, are indicated the mean divergence dates (in millions years ago; Mya) retrieved using either the time of divergence between *Carcharhinus* and *Sphyrna* genera (38 Mya; left) or the closure of the Isthmus of Panama separating Atlantic and Pacific populations (3.1–3.5 Mya; right)

The concatenated sequences *CR‐nd4‐cytb* (*N* = 218, fragment of 2,516 bp) resolved 36 haplotypes with an overall haplotype diversity of 0.93 ± 0.00 and a nucleotide diversity of 0.00551 ± 0.00002. No partitioning scheme was detected within the concatenated sequence, and the HKY85 + I model of substitution was selected with the BIC criterion. For both calibration strategies, Bayesian analyses of the concatenated mitochondrial sequence *CR‐nd4‐cytb* produced topologies with high support at most internal nodes and showed good convergence and mixing, with ESS above 200 (Table S4.1 in Appendix S4). For each analysis, similar lineages were strongly supported, with a first splitting event between the WA and both the WIO and WP populations, a second splitting event between the WIO and WP populations, and a third splitting event into two lineages within the WIO (Figure 3).

The calibration of the tree with the divergence date between *Sphyrna* and *Carcharhinus* genera, 38 Mya, provided a divergence rate between lineages per million years of 0.61% (95% confidence interval = [0.12, 1.23]). Using this calibration, populations from WA and both the WIO and WP diverged at 1.23 Mya [0.22, 4.27], while WIO and WP populations diverged at 0.75 Mya [0.05, 1.22]. The calibration of the tree with the date of closure of the Isthmus of Panama, 3.1–3.5 Mya, provided a divergence rate between lineages per million years of 0.24% [0.14, 0.36] and a divergence date of 1.69 Mya [0.75, 2.69] between WIO and WP populations. The mean of the two divergence rates was estimated, providing a mean substitution rate per site per year of 4.23 × 10^−9^ [1.14 × 10^−9^, 1.17 × 10^−8^].

**Figure 4:**
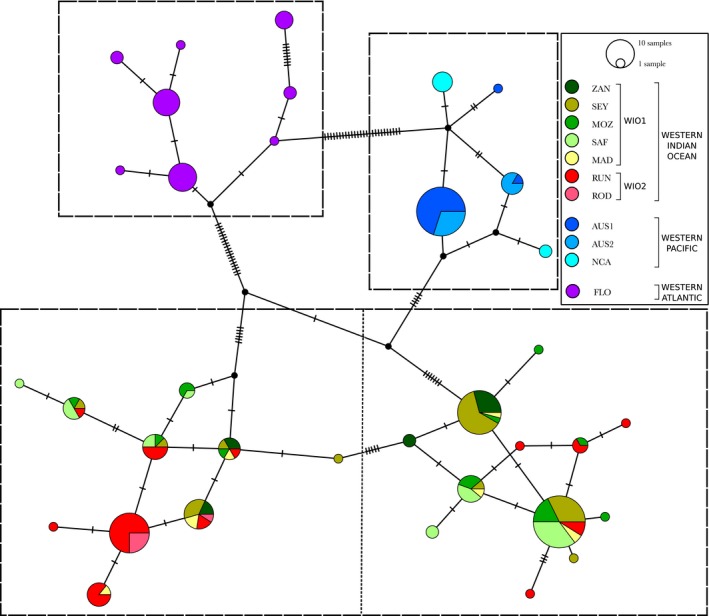
TCS statistical parsimony network of 36 bull sharks mitochondrial concatenated sequence *CR‐nd4‐cytb* haplotypes. Each circle represents a haplotype and each segment, a mutation. Boxes and the dotted line separating the Western Indian Ocean in two groups demarcate lineages discussed in the text (WIO1/WIO2). Circle size is proportional to the number of individuals harboring each haplotype, and colors correspond to sampling locations (WIO1: ZAN, Zanzibar; SEY, Seychelles; MOZ, Mozambique; SAF, South Africa; MAD, Madagascar; WIO2: RUN, Reunion Island; ROD, Rodrigues Island; AUS1, Clarence River, Australia; AUS2, Sydney Harbour, Australia; NCA, New Caledonia; FLO, Florida)

The TCS statistical parsimony network built from the *CR‐nd4‐cytb* dataset retrieved the same lineages as the phylogenetic tree, and highlighted the absence of shared haplotypes among lineages retrieved in each region. Twenty‐three haplotypes were identified in the WIO, five in the WP, and eight in the WA (Figure 4). Furthermore, the two lineages retrieved in the WIO seemed to correspond to the locations sampled along or near the African east coast (i.e., WIO1: Zanzibar, Seychelles, Mozambique, South Africa, and Madagascar) and to the Mascarene Islands (i.e., WIO2: Reunion Island and Rodrigues Island), despite some shared haplotypes.

Haplotype and nucleotide diversities were globally weaker in the WP (*h* = 0.51 ± 0.01 and *π* = 0.00056 ± 0.00002) than in the WIO and in the WA (WIO: *h* = 0.88 ± 0.00 and *π* = 0.00191 ± 0.00000; WA: *h* = 0.80 ± 0.01 and π = 0.00131 ± 0.00005). Within the WIO, *h* ranged from 0.33 ± 0.01 to 0.93 ± 0.03 and *π* from 0.00013 ± 0.00007 to 0.00212 ± 0.00046, both for Rodrigues Island and Madagascar, respectively. Within the WP, Clarence River (AUS1) showed the lowest values (*h* = 0.17 ± 0.02 and *π* = 0.00024± 0.00005) and Sydney Harbour (AUS2), the highest (*h* = 0.49 ± 0.02 and *π* = 0.00059± 0.00011; Table 1). Geographic distributions of all haplotypes are indicated in Appendix S5.

### 3.2 Genetic clustering

Structure clustering analysis suggested that the genetic structure is best explained by two clusters. For the microsatellite dataset without the LOCPRIOR model, a clear clustering was observed at *K* = 2 between samples from the WA and those from both the WIO and WP (Appendix S6a). For increasing *K* values, one cluster was identified in the WA, and subsequent clusters were represented in similar proportions in each individual from the WIO and the WP. When removing samples from the WA, for *K *= 2, each individual was equally assigned to both clusters, confirming the presence of only one genetic cluster (Appendix S6a).

**Figure 5:**
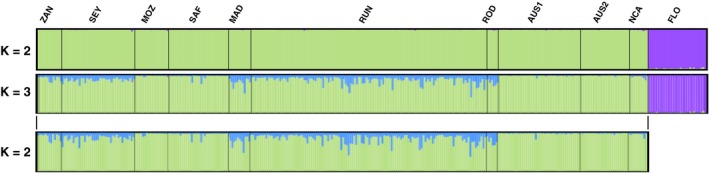
Average probability of membership (*y*‐axis) of bull shark individuals (*N* = 357, *x‐*axis) using 25 microsatellites, assuming correlated allele frequencies and admixture as performed by Structure with the LOCPRIOR model. Only major modes for *K* varying from two to three are presented. ZAN, Zanzibar; SEY, Seychelles; MOZ, Mozambique; SAF, South Africa; MAD, Madagascar; RUN, Reunion Island; ROD, Rodrigues Island; AUS1, Clarence River, Australia; AUS2, Sydney Harbour, Australia; NCA, New Caledonia; FLO, Florida

Using the LOCPRIOR model on the microsatellite dataset, all samples included, similar results were retrieved for *K* = 2 (Figure 5 and Appendix S6b). For increasing *K*, each newly identified cluster was found to be largely uninformative, with individual membership proportions in new clusters low. Similar results were retrieved for analyses using the microsatellite and mitochondrial datasets, both without and with the LOCPRIOR model (Appendix S6c,d).

**Figure 6:**
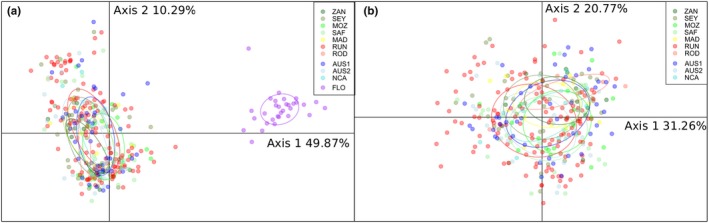
Bull shark scatter plot output from a DAPC from microsatellites using the first and second components (a) all sampling locations kept and (b) removing FLO. Dots represent individuals with sampling locations in colors (ZAN, Zanzibar; SEY, Seychelles; MOZ, Mozambique; SAF, South Africa; MAD, Madagascar; RUN, Reunion Island; ROD, Rodrigues Island; AUS1, Clarence River, Australia; AUS2, Sydney Harbour, Australia; NCA, New Caledonia; FLO, Florida)

The DAPC performed on microsatellites confirmed the clear clustering between the WA and both the WIO and WP with the first axis explaining 49.87% of total inertia. Locations from the WIO and the WP were not tightly grouped, with the second axis explaining 10.29% of total inertia, and ellipses for each location still overlapped (Figure 6a). When removing samples from Florida, ellipses of each location remained overlapped, the first axis explaining 31.26% and the second 20.77% of total inertia (Figure 6b).

### 3.3 Genetic differentiation

AMOVAs were conducted with the previously obtained clusters (microsatellites: WA and WIO/WP; mtDNA: WIO1, WIO2, WP, and WA) as first level of structuration. Percentages of variation associated with clusters were 26.35% and 81.61% for the microsatellite and the mitochondrial datasets, respectively. The weakest level of differentiation was observed among locations within clusters, with percentages of variation of 0.54% and 1.68% for the microsatellites and mtDNA, respectively (Appendix S7). Pairwise *F*_ST_ and *D*_est_ among locations from the WIO and the WP were weak (*F*_ST_ = [0.000, 0.047] and *D*_est_ = [0.000, 0.039]; higher values found for Rodrigues Island may be biased by low sample size), while the ones between all locations and WA were high (*F*_ST_ = [0.252, 0.335], all *p* < .001 after FDR correction and *D*_est_ = [0.313, 0.360], all *p* < .01 after FDR correction; Table 2). Similarly, pairwise Φ_ST_ values for the mitochondrial concatenated dataset were high among locations from the three regions (Φ_ST_ = [0.776, 0.929], all *p* < .001 after FDR correction; Table 2), and within the WIO, pairwise Φ_ST_ values were higher between locations from the Mascarene Islands (Reunion Island and Rodrigues Island) and the other locations that are along or near the African east coast (Zanzibar, Mozambique, South Africa, Seychelles). Φ_ST_ values varied from 0.346 (South Africa/Reunion Island) to 0.623 (Seychelles/Rodrigues Island; all *p* < .001 after FDR correction; Table 2). Within the WP, pairwise Φ_ST_ values varied from 0.193 to 0.509 and were all significantly different from zero after FDR correction (Table 2).

**Table 2:** Bull shark genetic differentiation between sampling locations (ZAN, Zanzibar; SEY, Seychelles; MOZ, Mozambique; SAF, South Africa; MAD, Madagascar; RUN, Reunion Island; ROD, Rodrigues Island; AUS1, Clarence River, Australia; AUS2, Sydney Harbour, Australia; NCA, New Caledonia; FLO, Florida) estimated for (a) microsatellite loci with Weir and Cockerham's *F*_ST_ (lower‐left matrix) and Jost's *D*_est_. (upper‐right matrix) estimates and (b) the mitochondrial dataset *CR‐nd4‐cytb* with Weir and Cockerham's Φ_ST_ (lower‐left matrix)

	ZAN	SEY	MOZ	SAF	MAD	RUN	ROD	AUS1	AUS2	NCA	FLO
(a) Microsatellites											
ZAN (13)	–	0.000	0.006	0.004	0.000	0.006	**0.039** *	0.000	0.002	0.006	**0.359** **
SEY (39)	0.000	–	0.005	0.006	0.000	0.003	0.026	0.006	0.006	0.016	**0.350** **
MOZ (18)	0.013	0.010	–	0.000	0.008	0.006	0.02	0.008	0.001	0.000	**0.313** **
SAF (32)	0.011	**0.009** *	0.000	–	0.005	0.003	0.031	0.002	0.005	0.008	**0.320** **
MAD (12)	0.000	0.000	0.010	0.007	–	0.000	0.014	0.001	0.007	0.019	**0.360** **
RUN (126)	0.010	0.004	**0.010** *	0.004	0.000	–	**0.032** *	**0.005** *	0.004	0.017	**0.329** **
ROD (6)	0.035	0.025	0.023	**0.030** *	0.009	**0.030** *	–	**0.034** *	0.031	0.056	**0.357** **
AUS1 (44)	0.004	**0.008** *	0.008	0.001	0.007	**0.008** **	**0.034** *	–	0.005	0.015	**0.340** **
AUS2 (26)	0.009	**0.010** *	0.001	0.005	0.015	0.006	0.033	0.004	–	0.015	**0.351** **
NCA (10)	0.005	0.009	0.000	0.001	0.012	0.009	**0.047** *	0.005	0.009	–	**0.333** **
FLO (31)	**0.317** ***	**0.285** ***	**0.272** ***	**0.265** ***	**0.300** ***	**0.252** ***	**0.335** ***	**0.271** ***	**0.297** ***	**0.287** ***	–
(b) *CR‐nd4‐cytb*											
ZAN (13)											
SEY (36)	0.027										
MOZ (18)	0.119	0.022									
SAF (25)	**0.184** *	**0.090** *	0.000								
MAD (8)	0.031	0.105	0.058	0.091							
RUN (38)	**0.396** ***	**0.435** ***	**0.354** ***	**0.346** ***	0.108						
ROD (6)	**0.618** ***	**0.623** ***	**0.581** ***	**0.551** ***	**0.342** *	0.057					
AUS1 (23)	**0.887** ***	**0.850** ***	**0.870** ***	**0.856** ***	**0.890** ***	**0.868** ***	**0.973** ***				
AUS2 (14)	**0.836** ***	**0.816** ***	**0.823** ***	**0.815** ***	**0.829** ***	**0.840** ***	**0.943** ***	**0.193** *			
NCA (7)	**0.804** ***	**0.805** ***	**0.797** ***	**0.794** ***	**0.776** ***	**0.821** ***	**0.928** ***	**0.509** ***	**0.234** *		
FLO (30)	**0.883** ***	**0.887** ***	**0.882** ***	**0.881** ***	**0.874** ***	**0.883** ***	**0.907** ***	**0.929** ***	**0.909** ***	**0.895** ***	

Note

Test significances were assessed after FDR correction and values significantly different from zero are indicated in bold. The number of individuals used for the analyses are indicated in parentheses.

* *p* < .05.

** *p* < .01.

*** *p* < .001.

### 3.4 Demographic history

#### 3.4.1 Neutrality tests

Considering the concatenated mitochondrial dataset, no evidence of any historical population expansions or contractions was found with tests of selective neutrality, either by considering all locations separately or by grouping them in the clusters identified (all Tajima's *D* and Fu's *F*_S_ not significantly different from zero; Appendix S8).

#### 3.4.2 Bayesian analyses using both microsatellite and mtDNA data

The PCAs on the space of the summary statistics and the analysis of the distribution of each summary statistics revealed that all scenarios could produce simulated datasets mirroring the observed dataset. On the PCAs of the summary statistics, the point representing the observed dataset fell within the cloud of points representing the simulated ones (Appendix S9). Also, most often the observed summary statistics fell well within the distribution obtained from the simulations (Appendix S10).

In all 10 replicates, Scenario 3 had the highest percentage of votes with 38.98% ± 0.97, while Scenario 1 the lowest with 5.73% ± 0.73 (Table 3). Performing Tukey's post hoc tests, we confirmed that Scenario 3 had a significantly higher percentage of votes compared to all others (all *p* < .001), while no significant differences were found between Scenario 2 and Scenario 4 (*p* = .15). Parameter values were thus estimated using data simulated under Scenario 3 only.

**Table 3:** Model choice procedure of the ABC random forest method used to compare demographic scenarios of bull shark populations from the Western Indian Ocean (RUN, Reunion Island) and the Western Pacific (AUS1, Clarence River, Australia)

Scenario	Votes (%)	Posterior probability	Prior error rate
Scenario 1	5.73 (0.73)		
Scenario 2	26.08 (1.11)		
Scenario 3	38**.**98 (0**.**97)	0.68 (0.01)	0.35 (0.00)
Scenario 4	29.2 (1.17)		

Note

Values are averaged over 10 replicate analyses and in parentheses are the standard errors. In bold is the best scenario selected.

Using 1,000 pseudo‐observed datasets, we found that for effective population sizes, the estimation procedure had very low bias and good precision over the whole prior range with low NMSE values, ranging from 0.02 to 0.03 for contemporary populations and 0.14 to 0.16 for the ancestral unsampled population (Table 4 and Appendix S11). Using these estimations, effective population sizes in number of genes estimated from the microsatellite data ranged from 7,090 (95% CI = [775; 62,928]) for AUS1 to 7,960 (95% CI = [1,016; 53,146]) for RUN, corresponding to effective population sizes of 3,545 and 3,980 individuals for AUS1 and RUN populations, respectively Those estimates using mtDNA varied from 376 genes (95% CI = [106; 4,728]) for AUS1 to 1,820 (95% CI = [494; 47,793]) for RUN. Other parameters were less well resolved (Appendix S12), and values will not be interpreted.

**Table 4:** Characteristics of posterior distributions of bull shark effective population size (*N*_e_) of contemporary populations estimated with ABC random forest method

Parameter	log10(*N*_e_(sat)_RUN_)	log10(*N*_e_(sat)_AUS1_)	log10(*N*_e_(seq)_RUN_)	log10(*N*_e_(seq)_AUS1_)
Expectation	3.89 (0.01)	3.85 (0.00)	3.37 (0.01)	2.62 (0.03)
Median	3.90 (0.03)	3.86 (0.01)	3.26 (0.02)	2.57 (0.03)
Variance	0.06 (0.02)	0.07 (0.03)	0.17 (0.05)	0.16 (0.02)
2.5% quantile	3.01 (0.00)	2.89 (0.03)	2.69 (0.03)	2.03 (0.01)
97.5% quantile	4.73 (0.01)	4.8 (0.01)	4.68 (0.02)	3.67 (0.04)
OOB‐MSE	0.05 (0.00)	0.06 (0.00)	0.10 (0.00)	0.11 (0.00)
NMSE	0.02	0.02	0.03	0.03
NMAE	0.06	0.06	0.08	0.08
Mean relative CI	0.30	0.32	0.39	0.40
Median relative CI	0.30	0.32	0.38	0.39

Note

*N*_e_ is expressed in number of genes; *N*_e_(sat), effective population size estimated using microsatellite data; *N*_e_(seq), effective population size estimated using mtDNA; OOB‐MSE, out‐of‐bag mean square error; NMSE, normalized mean square error; NMAE, normalized mean absolute error; CI, 95% confidence interval.

## 4 DISCUSSION

Using a combination of markers following different models of evolution and appropriate inference methods may help reach a better understanding of genetic structure and connectivity. Here, hierarchical sampling (inter‐ and intra‐ocean basins) and the use of both mtDNA sequences and microsatellite markers allowed us to test for the existence of migration between populations and to estimate effective population sizes of the bull shark. Strong genetic differentiation at both marker sets was observed between bull shark populations from the Western Atlantic and those of both the Western Indian Ocean and the Western Pacific (hereafter designated by Western Indian Ocean/Western Pacific), suggesting an absence of migration between the two regions. There was high differentiation in mtDNA in sharks from the Western Indian and Western Pacific Oceans, with no shared haplotype between the two regions. In contrast, low differentiation was inferred from microsatellite data. Within the Western Indian Ocean and the Western Pacific separately, this contrast was considerably less, suggesting some connectivity and/or high effective population sizes within each of these regions.

### 4.1 An ancient divergence between the Atlantic and the Western Indian Ocean/Western Pacific

Both mtDNA sequences and microsatellite markers showed high differentiation, suggesting a complete absence of gene flow between the Western Atlantic and the Western Indian Ocean/Western Pacific since their divergence. This result is congruent with previous research on bull shark using microsatellites, which identified three isolated genetic clusters, one in Indo‐Australia, one in Fiji, and one in the Atlantic Ocean (Testerman, 2014). This possibly long‐dating genetic divergence may have enabled the emergence of biological differences between Atlantic Ocean bull shark populations on one side, and those of the Indian and Pacific Oceans (Indian/Pacific Oceans) on the other. In the Indian/Pacific Oceans, individuals are larger, both in terms of maximum length (Blaison et al., 2015) and size at maturity (Cliff & Dudley, 1991) than those from the Gulf of Mexico (Branstetter & Stiles, 1987; Cruz‐Martinez, Chiappa‐Carrara, & Arenas‐Fuentes, 2005).

Divergence times were inferred based on a molecular clock estimate and should thus be regarded as qualitative indicators, rather than precise values. The use of the divergence between *Sphyrna* and *Carcharhinus* genera, or of the Isthmus of Panama closure as the divergence date between the Atlantic and the Indian/Pacific bull shark populations, yielded mutation rates similar to those observed in other shark species using several different fossil records (Duncan et al., 2006; Gubili et al., 2014; Karl, Castro, & Garla, 2012; Schultz et al., 2008). Using two different calibration dates, we estimated the divergence time of the Atlantic and the Indian/Pacific populations to be ca. 1.23 Mya [0.22 Mya–4.27 Mya], between the end of the Pliocene and the beginning of the Pleistocene. Divergence between these bull shark populations may be due to two biogeographical events: (a) the closure of the Isthmus of Panama, which occurred 3.1–3.5 Mya, and was important in shaping the current distribution of many species and genera by closing the link between the Eastern Pacific and the Western Atlantic (Briggs, 1995; Coates et al., 2004), and (b) the formation of the Benguela Upwelling System (~2 Mya), a cold water oceanographic system running along the west coast of South Africa and Namibia (Briggs, 1995) that restricts the mixing of tropical species populations between the Atlantic and the Indian Oceans via the southern tip of Africa (see Gaither, Bowen, Rocha, and Briggs (2016) for a review). Nevertheless, despite a small sample size in the Eastern Pacific (*n* = 5), Testerman (2014) identified only one cluster that grouped bull shark samples from the Eastern Pacific and the Western Atlantic, suggesting that bull shark migration might have occurred after the Isthmus of Panama closure through the Panama Canal. Such a scenario is possible since bull sharks are known to travel many hundreds of kilometers upstream in freshwater rivers and lakes (Heupel & Simpfendorfer, 2008; Thorson, 1976). The lack of samples from the Eastern Pacific did not allow us to test this hypothesis or the presence of any relationships between animals from the Eastern and the Western Pacific. Yet, populations from these two regions might be genetically structured because of the East Pacific Barrier, in place since 65 Mya (Grigg & Hey, 1992). This biogeographical barrier is characterized by depths over 5,000 m over a wide oceanic distance (~7,000 km), limiting longitudinal dispersal across the Pacific Ocean (Briggs, 1995). Nevertheless, some gene flow among these three regions may have occurred after the formation of the East Pacific Barrier, via the southern tip of Africa, before the formation of the Benguela Upwelling System.

The Benguela Upwelling System may be more constraining than the closure of the Isthmus of Panama for the bull shark, which is more sensitive to cold temperatures than species for which some gene flow after the formation of this current has been highlighted (e.g., tiger shark *Galeocerdo cuvier* [Bernard et al., 2016], dusky shark *Carcharhinus obscurus* [Benavides et al., 2011], or scalloped hammerhead shark *Sphyrna lewini* [Duncan et al., 2006]). Bull sharks remain in warmer waters, favoring temperatures of 24–26°C (Smoothey et al., 2016), and found less frequently in waters less than 18°C (Brunnschweiler et al., 2010; Lea et al., 2015; Matich & Heithaus, 2012). The formation of the Benguela Upwelling System may have disrupted the migratory behavior of bull sharks and led to the divergence of the Atlantic and Indian Ocean populations. Additional samples from the Eastern Pacific and the Southern Atlantic (both Eastern and Western) are needed to further investigate the worldwide phylogeography of the bull shark.

### 4.2 Negligible gene flow between Western Indian Ocean and Western Pacific

We observed a high differentiation at mtDNA sequences and a low differentiation at microsatellite markers between the Western Indian and the Western Pacific Oceans, a finding that had not been identified in previous studies. For example, Testerman (2014) only used nuclear information and found an absence of genetic differentiation between the two regions, while Tillett et al. (2012) used both types of markers but only sampled Northern Australia. Such a pattern is actually common in animal species (reviewed in Toews & Brelsford, 2012) and has already been described between bull shark populations from the northwestern and the southwestern Atlantic (Karl et al., 2011). Mitochondria have a uniparental mode of transmission and are haploid, and their sequences have a much lower mutation rate than microsatellites loci. Higher differentiation of mtDNA sequences has often been interpreted as indicative of female philopatry, due to the maternal inheritance of mitochondria and the biparental inheritance of nuclear microsatellite markers (e.g., Bernard et al., 2016; Karl et al., 2011; Pardini et al., 2001; Portnoy et al., 2015). But sex‐biased dispersal is not the only possible cause of a higher differentiation in mtDNA sequences as compared to microsatellite markers. In addition to their difference in modes of evolution, nonpanmictic mating systems may affect differentially the levels of differentiation at both types of markers. ABC random forest procedure, which is regarded as one of the most precise Bayesian methods to identify demographic histories (Fraimout et al., 2017; Pudlo et al., 2016; Raynal et al., 2017), offers a mean to formally test for the evolutionary forces underlying genetic population structure, including migration regimes. To do so and to account for sex‐biased dispersal, we independently estimated migration rates and effective sizes for both types of markers. Analyses revealed that the scenario with no gene flow between the Western Indian Ocean and the Western Pacific populations since their isolation best explained the observed data. Indeed, while scenarios with migration were designed to allow sex‐biased dispersal, they were chosen significantly less to explain the observed data than the scenario with no migration over 10 independent replicate analyses. This may reflect either an absence of gene flow or dispersal events that are rare enough not to be detected. For populations of large sizes (*N*_e_ > 10^3^), rare effective dispersal events may be sufficient to homogenize allelic frequencies, leading to *F*_ST_ estimates nonsignificantly different from zero (in the order of 10^−3^) while maintaining high mitochondrial differentiation (Hauser & Carvalho, 2008; Mariani & Bekkevold, 2014).

To increase juvenile survival, females may exhibit high fidelity to their breeding areas and nurseries, which are typically good foraging areas and offer protection from large predators (Branstetter, 1990; Castro, 1993; Heupel, Carlson, & Simpfendorfer, 2007; Springer, 1967). These breeding sites are sometimes the same as the natal places of females, as these latter represent suitable habitats for parturition (Heupel et al., 2007; Hueter et al., 2005). Female philopatry to nursery areas has notably been demonstrated in the lemon shark *Negaprion brevirostris* in the Bahamas by reconstructing parental genotypes (microsatellites) through sampling juveniles in specific nurseries over several decades: Some females returned to the nursery to give birth, sometimes 14 to 17 years after being born (Feldheim et al., 2013). In contrast, males may exhibit roaming behaviors and undertake migration, possibly to avoid inbreeding depression, and demographic and environmental stochasticity, especially in polygynous systems (Henry, Coulon, & Travis, 2016), as may occur for the bull shark (A. Pirog, personal communication). It is thus possible that female philopatry to nursery areas also occurs in the bull shark as hypothesized in the Western Atlantic (Karl et al., 2011) and in Australia (Tillett et al., 2012). Furthermore, no direct evidence of bull sharks moving between the Western Indian Ocean and the Western Pacific has been documented using satellite tracking or conventional tagging. While it may be due to relatively small sample sizes, it may also illustrate the absence, or at least extremely low occurrence, of bull shark migration across the Indian and Pacific Oceans.

Hypotheses of female philopatry in the bull shark, as well as the absence of known migration of bull sharks between the two oceans, support the absence of gene flow evidenced by the ABC‐RF analyses. A better knowledge of the mutational models of the two markers types in the bull shark, as well as genome‐wide analyses, would nevertheless be useful to confirm this absence of gene flow.

The negligible dispersal between the Western Indian Ocean and the Western Pacific may result from environmental barriers. Mitochondrial analyses indicated a divergence date of 0.75–1.69 Mya between Western Indian and Western Pacific bull shark populations. With as many as 20 glacial periods during the Pleistocene, each lasting approximately 100,000 years, followed by shorter interglacial periods of about 10,000 years (Dawson, 1992; Martinson et al., 1987), fluctuations in sea levels were as great as 100 m during this time period (Shackleton, 1987). These fluctuations may have changed the distribution of shallow, near‐shore habitats used by bull sharks and modified their movement patterns along the coasts, especially in Indonesia, possibly explaining the divergence between bull shark populations from the Western Indian Ocean and the Western Pacific. Indeed, several studies on chondrichthyan species have shown greater population subdivision between Indonesia and Northern Australia than within Australian waters (Dudgeon, Broderick, & Ovenden, 2009; Ovenden, Kashiwagi, Broderick, Giles, & Salini, 2009). It is possible that the deep waters of the Timor Trench (2,000–3,000 m) and the strong Indonesian through‐flow current along the Makassar and Lombok Straits induced the genetic subdivisions observed between Indonesian and Australian waters (Dudgeon et al., 2012, 2009; Ovenden et al., 2009), and thus limits gene flow between the Indian and Pacific Oceans.

### 4.3 Gene flow within the Western Indian Ocean and within the Western Pacific

Low genetic differentiation was shown both within the Western Indian Ocean and within the Western Pacific, regardless of the markers used (microsatellites or mtDNA). To date, a limited number of tracking studies have explored long‐distance movements of adult bull sharks, but each has highlighted the capability of bull sharks to undertake long‐distance coast‐wise migrations (up to 1,770 km; Carlson, Ribera, Conrath, Heupel, & Burgess, 2010; Daly et al., 2014; Espinoza, Heupel, Tobin, & Simpfendorfer, 2016; Espinoza, Lédée, Simpfendorfer, Tobin, & Heupel, 2015; Heupel et al., 2015) and across hundreds of kilometers of open ocean (Soria et al., 2015). As such, long‐distance migration of adult bull sharks may genetically link ecosystems within these regions. Each movement study also highlights the fidelity of bull sharks to specific sites at discrete times, as shown in Reunion Island (Blaison et al., 2015), in New Caledonia (Werry & Clua, 2013), in Australia (Heupel et al., 2015), in Fiji, and in the Bahamas (Brunnschweiler & Baensch, 2011; Brunnschweiler et al., 2010). Thus, previous tracking studies and the low genetic differentiation from the present study suggest that individuals may disperse on a regular basis among locations within each of these regions. Nevertheless, slightly higher mitochondrial differentiation values were retrieved among locations separated by deep‐water expenses, such as the Mascarene Islands (WIO1) and locations along the Eastern African coast (WIO2), or among locations of the Eastern Australian coast and New Caledonia. These higher values may reflect some level of female philopatry to nursery areas at the described spatial scale. Indeed, even if some mitochondrial haplotypes are shared between these locations, as samples analyzed in this study were taken from sharks fished or caught opportunistically, the geographic location assigned to each individual does not reflect necessarily its nursery or natal site. Hence, the shared haplotypes potentially reflect female (and also male) movements between two stays (possibly lengthy ones) in their birthing and/or natal nurseries. As an illustration, a gravid female bull shark satellite‐tagged in the Seychelles traveled 2,000 km, to the southeast coast of Madagascar, where it remained in shallow waters for several days, before returning, no longer in a gravid condition to the Seychelles (Lea et al., 2015), suggesting this female may have given birth in Madagascar (perhaps its natal site) and therefore undertakes long‐distance movements between Madagascar and the Seychelles. However, no direct evidence of female philopatry to nursery sites has been documented for bull sharks. This would require the sampling of juveniles in nurseries for parentage analyses as direct observation of parturition is highly unlikely, especially for tagged females, due to the turbid nature of estuaries and frequency of occurrence.

### 4.4 Effective population sizes

Changes in population size were not detected with neutrality tests performed with mitochondrial data. Estimates of effective population sizes (*N*_e_) from the microsatellite dataset were ca. 3,000–4,000 individuals for the Western Indian Ocean and the Western Pacific. We obtained much lower estimates from the mitochondrial dataset, for example, approximately 1,800 (approximately 1/4 the microsatellite estimation) and 380 (approximately 1/20 the microsatellite estimation) for the Western Indian Ocean and the Western Pacific, respectively. Nevertheless, 95% confidence intervals were large and those estimated with mtDNA nearly overlapped those estimated with microsatellite data. Under panmixia, a lower effective population size is expected for uniparentally inherited markers, compared with biparentally inherited ones (autosomal markers), and a deviation from that expectation may reflect sex‐biased dispersal patterns, social organization, or specific mating systems. Chesser and Baker (1996) showed that in panmictic populations and in systems with single paternity, the effective size of maternally and paternally inherited markers was one‐half of that of biparentally inherited markers and that social structure, sex‐biased dispersal, or different mating systems usually lower the effective size of autosomal markers while lowering or uppering maternally and paternally inherited markers. The bull shark has recently been shown to be a polyandrous species (Pirog et al., 2015). Sugg and Chesser (1994) showed that multiple paternity increases the effective sizes of diploid genes. However, because all the progeny will receive the maternally inherited genes from the female regardless the sire, multiple paternity should not affect the dynamics of the genes that are transmitted by the female (Chesser & Baker, 1996). Estimates of *N_e_* inferred using mtDNA may thus be more accurate than those estimated using microsatellite data.

Using the mismatch distribution of the mitochondrial control region, Tillett et al. (2012) estimated larger long‐term *N*_e_ for bull shark populations from Northern Australia (Western Pacific), with a *θ* value of 0.293 corresponding to an effective population size of 11,000–13,000 individuals. Comparatively, using 11 microsatellite loci, Testerman (2014) estimated long‐term *N*_e_ of populations from the Western Atlantic, the Indo‐Pacific, and Fiji to be ca. 100,000 genes, that is, 50,000 individuals. Karl et al. (2011) found similar estimates using the mitochondrial control region and five microsatellite loci separately for populations of the Northern and southwestern Atlantic, with long‐term *N*_e_ ranging from 148,000 to 214,200 individuals. The discrepancy between our microsatellite estimates and those of previous studies may be due to the higher number of loci we used, 25 versus 11 and 5, with the accuracy in the estimate of *θ* being proportional to the number of loci (Felsenstein, 2006; Pluzhnikov & Donnelly, 1996). It may also be due to the scale of the region studied, as our estimates were obtained using samples from one locality to represent an entire region. Our mitochondrial estimates were nevertheless lower than those previously inferred.

Estimates of effective population size using genetic markers are increasingly used for fisheries stock assessments (Ovenden et al., 2016). It has been postulated that an *N*_e_ of at least 500 individuals is needed for a population to adapt to environmental changes (Frankham et al., 2010) although others estimate that at least 5,000 breeding individuals may be required (Lande, 1995). Avoiding deleterious allele accumulation may require an *N*_e_ above 1,000 individuals (Frankham et al., 2010; Palstra & Ruzzante, 2008) and inbreeding depression may occur if *N*_e_ falls below 50 individuals (Frankham et al., 2010). Our estimates (3,000–4,000 with microsatellite data; 380–1,800 with mtDNA) are nearly in the same range as the basking shark *Cetorhinus maximus* (i.e., 8,200; Hoelzel et al., 2006), but lower than estimates for the lemon shark *N. brevirostris* (26,000 to 52,000 in the Atlantic) and the sicklefin lemon shark *Negaprion acutidens* (34,000 to 52,000 in the Western Pacific; Schultz et al., 2008), and much lower than for the tope shark, *Galeorhinus galeus* (138,000; Chabot & Allen, 2009). All of these species are considered either (a) globally Vulnerable on the IUCN Red List or (b) subjected to a loss of genetic diversity due to a bottleneck (e.g., basking sharks). This may therefore be the case for bull sharks, especially if taking into account mtDNA estimates, and populations may even be depleted. Obtaining more precise population estimates requires greater knowledge of the reproductive biology of the bull shark, notably the number of individuals that successfully reproduce in a generation (or reproductive cycle), the age at maturity, and the mating system (Ovenden et al., 2016).

## 5 CONCLUSION

Here, we highlight several key findings about the global population structure of bull sharks that will inform management and conservation issues:

1. The genetic isolation between bull shark populations from the Western Atlantic and from the Western Indian Ocean/Western Pacific implies that the Western Atlantic populations should be managed separately.
2. Low gene flow, and maybe even complete isolation, has also been evidenced between bull shark populations from the Western Indian Ocean and the Western Pacific, despite a low nuclear differentiation. It implies that these populations should also be managed separately. Understanding that low nuclear differentiation is not a guarantee of extant gene flow may have important implications for population management.
3. Within the Western Indian Ocean and within the Western Pacific, males and females are capable of undertaking long‐distance movements at this scale, with either (a) both sexes contributing to effective dispersal (i.e., gene flow) or (b) males contributing to effective dispersal and females exhibiting philopatry to their natal sites for mating and/or breeding. Thus, conservation and management programs (e.g., postattack culling programs) may be ineffective if implemented at a very localized local scale.
4. Estimates of the effective bull shark population size using mtDNA were lower than when using microsatellite data. Lower estimates may result from a complex reproductive system, or from significant frequency of multiple paternity in the bull shark. While the estimates remain comparable to other shark species, mtDNA estimates of effective population size may indicate depleted populations, and caution should be taken when implementing fisheries guidelines for this species.

## CONFLICT OF INTEREST

None declared.

## AUTHOR CONTRIBUTIONS

MCF, GC, EC, RD, MRH, JJK, PM, JEGN, AFS, AJT, PB, and SJ provided samples. AP, HM, and MCF did laboratory work. AP, HM, VR, AR, and AG analyzed the data. All authors contributed to the writing of the manuscript. HM and SJ designed research.

## Data Availability

Sample information and microsatellite genotypes: Dryad https://doi.org/10.5061/dryad.kp32qr6. GenBank Accession Numbers for mitochondrial sequences: *CR*, MN227021–MN227039; *cytb*, MN227040–MN227054; and *nd4*, MN227055–MN227067.
