# Supplementary material for: Population structure, connectivity, and demographic history of an apex marine predator, the bull shark Carcharhinus leucas
Source: Ecol Evol. 2019 Sep 30;9(23):12980–3000. doi: 10.1002/ece3.5597 (PMC6912899; doi:10.1002/ece3.5597)
Supplement: Supplementary file 1 [file ECE3-9-12980-s001.docx]

**Appendix A1.** Panels used to multiplex loci post-PCR and allelic ranges (in base pairs, without M13-tail).

| Locus name | Panel | Fluorochrome | Allelic range | Reference |
| --- | --- | --- | --- | --- |
| Cl03 | 1 | VIC | 104-112 | Pirog et al. 2015 |
| Cl01 | 1 | 6-FAM | 114-116 | Pirog et al. 2015 |
| Gc01 | 1 | VIC | 136-146 | Pirog et al. 2016 |
| Cl06 | 1 | PET | 148-150 | Pirog et al. 2015 |
| Cl14 | 1 | VIC | 211-213 | Pirog et al. 2015 |
| Cl11 | 1 | NED | 232-238 | Pirog et al. 2015 |
| Cl12 | 2 | 6-FAM | 103-113 | Pirog et al. 2015 |
| Cl10 | 2 | PET | 123-133 | Pirog et al. 2015 |
| Cl02 | 2 | NED | 141-145 | Pirog et al. 2015 |
| Cl08 | 2 | VIC | 146-158 | Pirog et al. 2015 |
| Cl17 | 2 | 6-FAM | 167-183 | Pirog et al. 2015 |
| Cpl166 | 2 | PET | 218-352 | Portnoy et al. 2006 |
| Cl13 | 3 | VIC | 106-140 | Pirog et al. 2015 |
| Cl09 | 3 | PET | 107-115 | Pirog et al. 2015 |
| Cl05 | 3 | NED | 137-141 | Pirog et al. 2015 |
| Cl07 | 3 | 6-FAM | 143-151 | Pirog et al. 2015 |
| Cl18 | 3 | 6-FAM | 201-221 | Pirog et al. 2015 |
| Cl16 | 4 | NED | 103-121 | Pirog et al. 2015 |
| Cl04 | 4 | 6-FAM | 124-130 | Pirog et al. 2015 |
| Cl20 | 4 | VIC | 134-182 | Pirog et al. 2015 |
| Cl19 | 4 | NED | 198-219 | Pirog et al. 2015 |
| Cl15 | 4 | PET | 290-298 | Pirog et al. 2015 |
| Ct05 | 5 | NED | 226-250 | Ovenden et al. 2006 |
| Ls24 | 5 | 6-FAM | 249-275 | Feldheim et al. 2001 |
| TIG10 | 5 | PET | 257-265 | Mendes et al. 2016 |
