# Supplementary material for: Population structure, connectivity, and demographic history of an apex marine predator, the bull shark Carcharhinus leucas
Source: Ecol Evol. 2019 Sep 30;9(23):12980–3000. doi: 10.1002/ece3.5597 (PMC6912899; doi:10.1002/ece3.5597)
Supplement: Supplementary file 2 [file ECE3-9-12980-s002.docx]

**Table A2.1.** Priors for the demographic parameters

| log_10_(*N*) | unif [2-8] |
| --- | --- |
| *t*_1_ | logunif [1-350000] |
| *t*_2_ | logunif [220-350000] |
| *µ_seq_* | unif [10^-8^-10^-6^] |
| *µ_sat_* | unif [10^-5^-10^-4^] |
| *m* | unif [0.01-0.2] |
| Constraint on parameters | *t*_1_ < *t*_2_ |

*N*, effective population sizes of all populations; *t*_1_, start and end of the isolation period; *t*_2_, time of divergence; *µ_seq_*, sequence mutation rate; *µ_sat_*, microsatellite mutation rate; unif, uniform distribution; logunif, log-uniform distribution; in brackets are minimal and maximal values
