# Supplementary material for: Population structure, connectivity, and demographic history of an apex marine predator, the bull shark Carcharhinus leucas
Source: Ecol Evol. 2019 Sep 30;9(23):12980–3000. doi: 10.1002/ece3.5597 (PMC6912899; doi:10.1002/ece3.5597)
Supplement: Supplementary file 3 [file ECE3-9-12980-s003.docx]

**Table A2.2.** Summary statistics of the observed datasets estimated for the ABC analyses. For microsatellites: *K*¸ mean number of alleles over loci; *H*, mean of Nei’s gene diversity; *MGW*, mean over loci of the modified Garza-Williamson index; *F_ST_*, pairwise microsatellite *F_ST_*; *DMUSQR*, mean delta mu-square (square difference in mean microsatellite allele length between pairs of populations). For the mitochondrial marker: *seqK*¸ mean number of alleles over loci; *seqH*, mean of Nei’s gene diversity; *Pi*, mean number of pairwise differences; *D*, Tajima’s *D*; *Fs*, Fu’s *Fs*; *seqF_ST_*, pairwise *F_ST_*.

| Microsatellites | RUN-AUS1 |
| --- | --- |
| *K_RUN_* | 4.56 |
| *K_AUS1_* | 4.04 |
| *H_RUN_* | 0.44 |
| *H_AUS1_* | 0.43 |
| *MGW_RUN_* | 0.46 |
| *NGW_AUS1_* | 0.39 |
| *F_ST_* | 0.01 |
| *DMUSQ* | 0.23 |
| Mitochondrial |  |
| *seqK_RUN_* | 12.00 |
| *seqK_AUS1_* | 3.00 |
| *seqH_RUN_* | 0.81 |
| *seqH_AUS1_* | 0.17 |
| *D_RUN_* | -0.07 |
| *D_AUS1_* | -1.82 |
| *Fs_RUN_* | -1.37 |
| *Fs_AUS1_* | 0.24 |
| *Pi_RUN_* | 3.78 |
| *Pi_AUS1_* | 0.60 |
| *seqF_ST_* | 0.87 |
