# Supplementary material for: Population structure, connectivity, and demographic history of an apex marine predator, the bull shark Carcharhinus leucas
Source: Ecol Evol. 2019 Sep 30;9(23):12980–3000. doi: 10.1002/ece3.5597 (PMC6912899; doi:10.1002/ece3.5597)
Supplement: Supplementary file 4 [file ECE3-9-12980-s004.docx]

**Appendix A3.** Summary statistics for each sampling location for the three mitochondrial markers used, the control region *CR* (923 bp), *nd4* (672 pb) and *cytb* (921 bp). ZAN, Zanzibar; SEY, Seychelles; MOZ, Mozambique; SAF, South Africa; MAD, Madagascar; RUN, Reunion Island; ROD, Rodrigues Island; AUS1, Clarence River, Australia; AUS2, Sydney Harbour, Australia; NCA, New Caledonia; FLO, Florida. *N_s_*, number of individuals sequenced; *H*, number of haplotypes, *h*, haplotype diversity; *S*, number of polymorphic sites; *π*, nucleotide diversity. In parentheses, are indicated standard errors.

| *CR* | ZAN | SEY | MOZ | SAF | MAD | RUN | ROD | AUS1 | AUS2 | NCA | FLO | TOT |
| --- | --- | --- | --- | --- | --- | --- | --- | --- | --- | --- | --- | --- |
| *N_s_* | 13 | 36 | 18 | 29 | 13 | 66 | 6 | 31 | 15 | 9 | 31 | 267 |
| *H* | 3 | 4 | 5 | 4 | 3 | 5 | 1 | 3 | 2 | 2 | 6 | 19 |
| *h* | 0.64  (0.03) | 0.41  (0.01) | 0.66  (0.02) | 0.70  (0.01) | 0.62  (0.02) | 0.37  (0.01) | 0.00  (0.00) | 0.18  (0.02) | 0.51  (0.02) | 0.50  (0.04) | 0.74  (0.01) | 0.80  (0.00) |
| *S* | 5 | 5 | 5 | 5 | 5 | 9 | 0 | 4 | 1 | 3 | 6 | 18 |
| *π* | 0.00259 (0.00047) | 0.00193 (0.00021) | 0.00235 (0.00036) | 0.00236 (0.00028) | 0.00292 (0.00052) | 0.00184 (0.00015) | 0.00000 (0.00000) | 0.00041 (0.00008) | 0.00056 (0.00014) | 0.00163 (0.00041) | 0.00208 (0.00024) | 0.00448 (0.00001) |
| *nd4* | ZAN | SEY | MOZ | SAF | MAD | RUN | ROD | AUS1 | AUS2 | NCA | FLO | TOT |
| *N_s_* | 13 | 39 | 18 | 30 | 15 | 48 | 6 | 31 | 14 | 10 | 31 | 255 |
| *H* | 2 | 6 | 7 | 5 | 4 | 6 | 2 | 2 | 2 | 2 | 1 | 13 |
| *h* | 0.46  (0.03) | 0.71  (0.01) | 0.74  (0.02) | 0.54  (0.02) | 0.76  (0.02) | 0.63  (0.01) | 0.33  (0.09) | 0.12  (0.01) | 0.49  (0.02) | 0.47  (0.04) | 0.00  (0.00) | 0.86  (0.00) |
| *S* | 1 | 5 | 6 | 4 | 3 | 6 | 1 | 1 | 1 | 1 | 0 | 22 |
| *π* | 0.00069 (0.00020) | 0.00152 (0.00019) | 0.00173 (0.00031) | 0.00166 (0.00023) | 0.00193 (0.00037) | 0.00131 (0.00015) | 0.0005 (0.00027) | 0.00019 (0.00006) | 0.00074 (0.0002) | 0.00069 (0.00236) | 0.00000 (0.00000) | 0.00834 (0.00003) |
| *cytb* | ZAN | SEY | MOZ | SAF | MAD | RUN | ROD | AUS1 | AUS2 | NCA | FLO | TOT |
| *N_s_* | 13 | 39 | 18 | 27 | 9 | 40 | 6 | 23 | 15 | 7 | 30 | 227 |
| *H* | 3 | 3 | 3 | 4 | 3 | 5 | 1 | 2 | 2 | 2 | 5 | 15 |
| *h* | 0.51  (0.04) | 0.39  (0.01) | 0.50  (0.02) | 0.56  (0.02) | 0.64  (0.04) | 0.57  (0.01) | 0.00  (0.00) | 0.09  (0.02) | 0.51  (0.02) | 0.48  (0.06) | 0.54  (0.02) | 0.80  (0.00) |
| *S* | 2 | 2 | 2 | 3 | 2 | 3 | 0 | 1 | 1 | 1 | 7 | 23 |
| *π* | 0.00081 (0.0002) | 0.00060 (0.00009) | 0.00058 (0.00013) | 0.00078 (0.00013) | 0.00115 (0.00032) | 0.00095 (0.00012) | 0.00000 (0.00000) | 0.00009 (0.00004) | 0.00056 (0.00014) | 0.00052 (0.00022) | 0.00165 (0.00021) | 0.00426 (0.00002) |
