# Supplementary material for: Population structure, connectivity, and demographic history of an apex marine predator, the bull shark Carcharhinus leucas
Source: Ecol Evol. 2019 Sep 30;9(23):12980–3000. doi: 10.1002/ece3.5597 (PMC6912899; doi:10.1002/ece3.5597)
Supplement: Supplementary file 5 [file ECE3-9-12980-s005.docx]

**Table A4.1.** Effective Sample Sizes (ESS) of the posterior distribution and of the mean rate of substitution.

| ESS | (a) | (b) |
| --- | --- | --- |
| Posterior distribution | 373 | 333 |
| Mean rate of substitution | 1901 | 7182 |
