# Supplementary material for: Population structure, connectivity, and demographic history of an apex marine predator, the bull shark Carcharhinus leucas
Source: Ecol Evol. 2019 Sep 30;9(23):12980–3000. doi: 10.1002/ece3.5597 (PMC6912899; doi:10.1002/ece3.5597)
Supplement: Supplementary file 6 [file ECE3-9-12980-s006.docx]

**Appendix A5.** Geographic distribution of mitochondrial concatenated sequence *CR‑nd4‑cytb* haplotypes found in bull sharks from the Western Indian Ocean (WIO), the Western Pacific (WP) and the Western Atlantic (WA). ZAN, Zanzibar; SEY, Seychelles; MOZ, Mozambique; SAF, South Africa; MAD, Madagascar; RUN, Reunion Island; ROD, Rodrigues Island; AUS1, Clarence River, Australia; AUS2, Sydney Harbour, Australia; NCA, New Caledonia; FLO, Florida.

|  | **WIO** | | | | | | | **WP** | | | **WA** |  |
| --- | --- | --- | --- | --- | --- | --- | --- | --- | --- | --- | --- | --- |
| **Haplotypes** | **ZAN** | **SEY** | **MOZ** | **SAF** | **MAD** | **RUN** | **ROD** | **AUS1** | **AUS2** | **NCA** | **FLO** | **TOT** |
| **H01** |  | 11 | 6 | 10 | 2 | 3 |  |  |  |  |  | 32 |
| **H02** | 7 | 15 | 1 |  | 1 |  |  |  |  |  |  | 24 |
| **H03** | 2 | 4 |  |  | 2 | 2 | 1 |  |  |  |  | 11 |
| **H04** |  | 1 | 3 | 4 | 1 |  |  |  |  |  |  | 9 |
| **H05** |  | 1 | 1 | 2 |  | 4 |  |  |  |  |  | 8 |
| **H06** |  |  |  |  | 1 | 6 |  |  |  |  |  | 7 |
| **H07** | 2 | 1 | 1 |  | 1 | 1 |  |  |  |  |  | 6 |
| **H08** |  | 1 | 1 | 3 |  | 1 |  |  |  |  |  | 6 |
| **H09** |  |  | 2 | 1 |  |  |  |  |  |  |  | 3 |
| **H10** |  |  | 1 |  |  | 2 |  |  |  |  |  | 3 |
| **H11** | 2 |  |  |  |  |  |  |  |  |  |  | 2 |
| **H12** |  |  |  | 2 |  |  |  |  |  |  |  | 2 |
| **H13** |  |  |  | 2 |  |  |  |  |  |  |  | 2 |
| **H14** |  | 1 |  |  |  |  |  |  |  |  |  | 1 |
| **H15** |  | 1 |  |  |  |  |  |  |  |  |  | 1 |
| **H16** |  |  | 1 |  |  |  |  |  |  |  |  | 1 |
| **H17** |  |  | 1 |  |  |  |  |  |  |  |  | 1 |
| **H18** |  |  |  | 1 |  |  |  |  |  |  |  | 1 |
| **H19** |  |  |  |  |  | 15 | 5 |  |  |  |  | 20 |
| **H20** |  |  |  |  |  | 1 |  |  |  |  |  | 1 |
| **H21** |  |  |  |  |  | 1 |  |  |  |  |  | 1 |
| **H22** |  |  |  |  |  | 1 |  |  |  |  |  | 1 |
| **H23** |  |  |  |  |  | 1 |  |  |  |  |  | 1 |
| **H24** |  |  |  |  |  |  |  | 21 | 9 |  |  | 30 |
| **H25** |  |  |  |  |  |  |  | 1 | 5 |  |  | 6 |
| **H26** |  |  |  |  |  |  |  |  |  | 5 |  | 5 |
| **H27** |  |  |  |  |  |  |  |  |  | 2 |  | 2 |
| **H28** |  |  |  |  |  |  |  | 1 |  |  |  | 1 |
| **H29** |  |  |  |  |  |  |  |  |  |  | 10 | 10 |
| **H30** |  |  |  |  |  |  |  |  |  |  | 9 | 9 |
| **H31** |  |  |  |  |  |  |  |  |  |  | 4 | 4 |
| **H32** |  |  |  |  |  |  |  |  |  |  | 2 | 2 |
| **H33** |  |  |  |  |  |  |  |  |  |  | 2 | 2 |
| **H34** |  |  |  |  |  |  |  |  |  |  | 1 | 1 |
| **H35** |  |  |  |  |  |  |  |  |  |  | 1 | 1 |
| **H36** |  |  |  |  |  |  |  |  |  |  | 1 | 1 |
| **TOT** | 13 | 36 | 18 | 25 | 8 | 38 | 6 | 23 | 14 | 7 | 30 | 218 |
