# Supplementary material for: Population structure, connectivity, and demographic history of an apex marine predator, the bull shark Carcharhinus leucas
Source: Ecol Evol. 2019 Sep 30;9(23):12980–3000. doi: 10.1002/ece3.5597 (PMC6912899; doi:10.1002/ece3.5597)
Supplement: Supplementary file 7 [file ECE3-9-12980-s007.pdf]

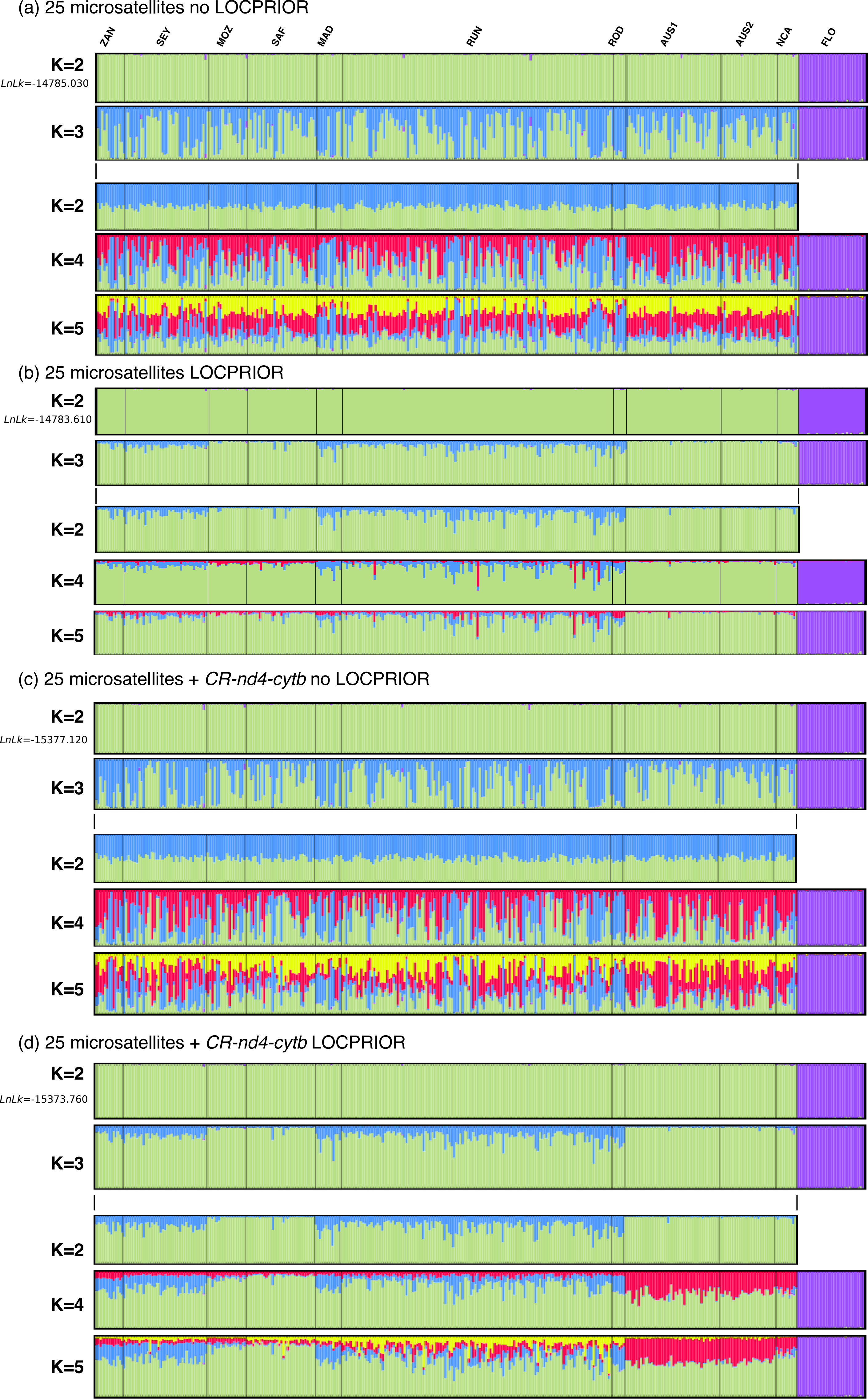
(a) 25 microsatellites no LOCPRIOR
ZAN
SEY
MOZ
SAF
MAD
RUN
ROD
AUS1
AUS2
NCA
FLO
K=2
LnLk=-14785.030
K=3
K=2
K=4
K=5
(b) 25 microsatellites LOCPRIOR
K=2
LnLk=-14783.610
K=3
K=2
K=4
K=5
(c) 25 microsatellites + CR-nd4-cytb no LOCPRIOR
K=2
LnLk=-15377.120
K=3
K=2
K=4
K=5
(d) 25 microsatellites + CR-nd4-cytb LOCPRIOR
K=2
LnLk=-15373.760
K=3
K=2
K=4
K=5
