# Supplementary material for: Population structure, connectivity, and demographic history of an apex marine predator, the bull shark Carcharhinus leucas
Source: Ecol Evol. 2019 Sep 30;9(23):12980–3000. doi: 10.1002/ece3.5597 (PMC6912899; doi:10.1002/ece3.5597)
Supplement: Supplementary file 8 [file ECE3-9-12980-s008.docx]

**Appendix A7.** Analyses of molecular variance performed for 25 microsatellite loci and three mitochondrial loci concatenated (*CR-nd4-cytb*). **: *P* < 0.01; ***: *P* < 0.001.

| **Microsatellites** |  | **Variance components** | **Percentage of variation** | **Φ** |
| --- | --- | --- | --- | --- |
| **Among genetic clusters** | FCT | 1.91 | 26.35 | 0.26*** |
| **(WIO/WP vs WA)** |  |  |  |  |
| **Among sampling locations within genetic clusters** | FSC | 0.04 | 0.54 | 0.01*** |
| **Within sampling locations** | FST | 5.31 | 73.12 | 0.27*** |
| **Total** |  | 7.26 |  |  |
| ***CR-nd4-cytb*** |  | **Variance components** | **Percentage of variation** | **Φ** |
| **Among genetic clusters** | FCT | 7.59 | 81.61 | 0.82*** |
| **(WIO1-WIO2-WP-WA)** |  |  |  |  |
| **Among sampling locations within genetic clusters** | FSC | 0.16 | 1.68 | 0.09*** |
| **Within sampling locations** | FST | 1.55 | 16.71 | 0.83*** |
| **Total** |  | 9.3 |  |  |
