# Supplementary material for: Population structure, connectivity, and demographic history of an apex marine predator, the bull shark Carcharhinus leucas
Source: Ecol Evol. 2019 Sep 30;9(23):12980–3000. doi: 10.1002/ece3.5597 (PMC6912899; doi:10.1002/ece3.5597)
Supplement: Supplementary file 9 [file ECE3-9-12980-s009.docx]

**Appendix A8.** *Carcharhinus leucas* demography statistics (Tajima’s *D* and Fu’s *F_S_*) for the concatenated mitochondrial sequence *CR-nd4-cytb* (ZAN, Zanzibar; SEY, Seychelles; MOZ, Mozambique; SAF, South Africa; MAD, Madagascar; RUN, Reunion Island; ROD, Rodrigues Island; AUS1, Clarence River, Australia; AUS2, Sydney Harbour, Australia; NCA, New Caledonia; FLO, Florida). All values were not significantly different from zero (*P*> 0.05).

|  | *D* | *F_S_* |
| --- | --- | --- |
| Western Indian Ocean | 0.740 | -2.725 |
| *African east coast (WIO1)* | 0.575 | -2.235 |
| ZAN | 1.520 | 2.737 |
| SEY | 0.502 | 0.116 |
| MOZ | 0.089 | -2.181 |
| MAD | 1.880 | -0.355 |
| SAF | 1.059 | 0.623 |
| *Mascarene Islands (WIO2)* | -0.261 | -1.409 |
| RUN | -0.068 | -1.373 |
| ROD | -0.933 | -0.003 |
| Western Pacific | -0.352 | 0.816 |
| AUS1 | -1.821 | 0.244 |
| AUS2 | 1.753 | 3.641 |
| NCA | 0.826 | 3.754 |
| Western Atlantic |  |  |
| FLO | 0.020 | 0.402 |
