# Supplementary figures and images for: Population structure, connectivity, and demographic history of an apex marine predator, the bull shark Carcharhinus leucas
Source: Ecol Evol. 2019 Sep 30;9(23):12980–3000. doi: 10.1002/ece3.5597 (PMC6912899; doi:10.1002/ece3.5597)

Prior checking

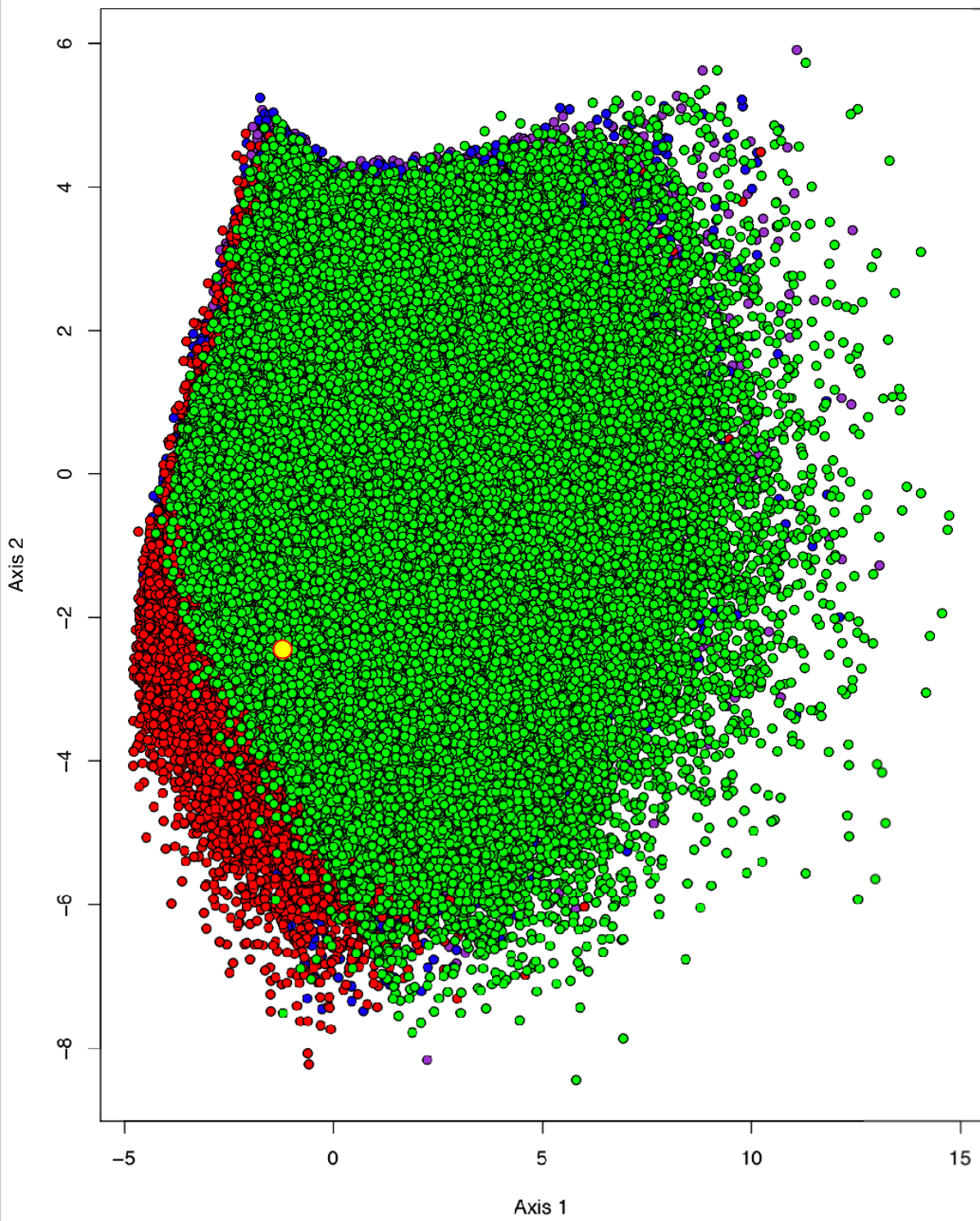

Prior checking

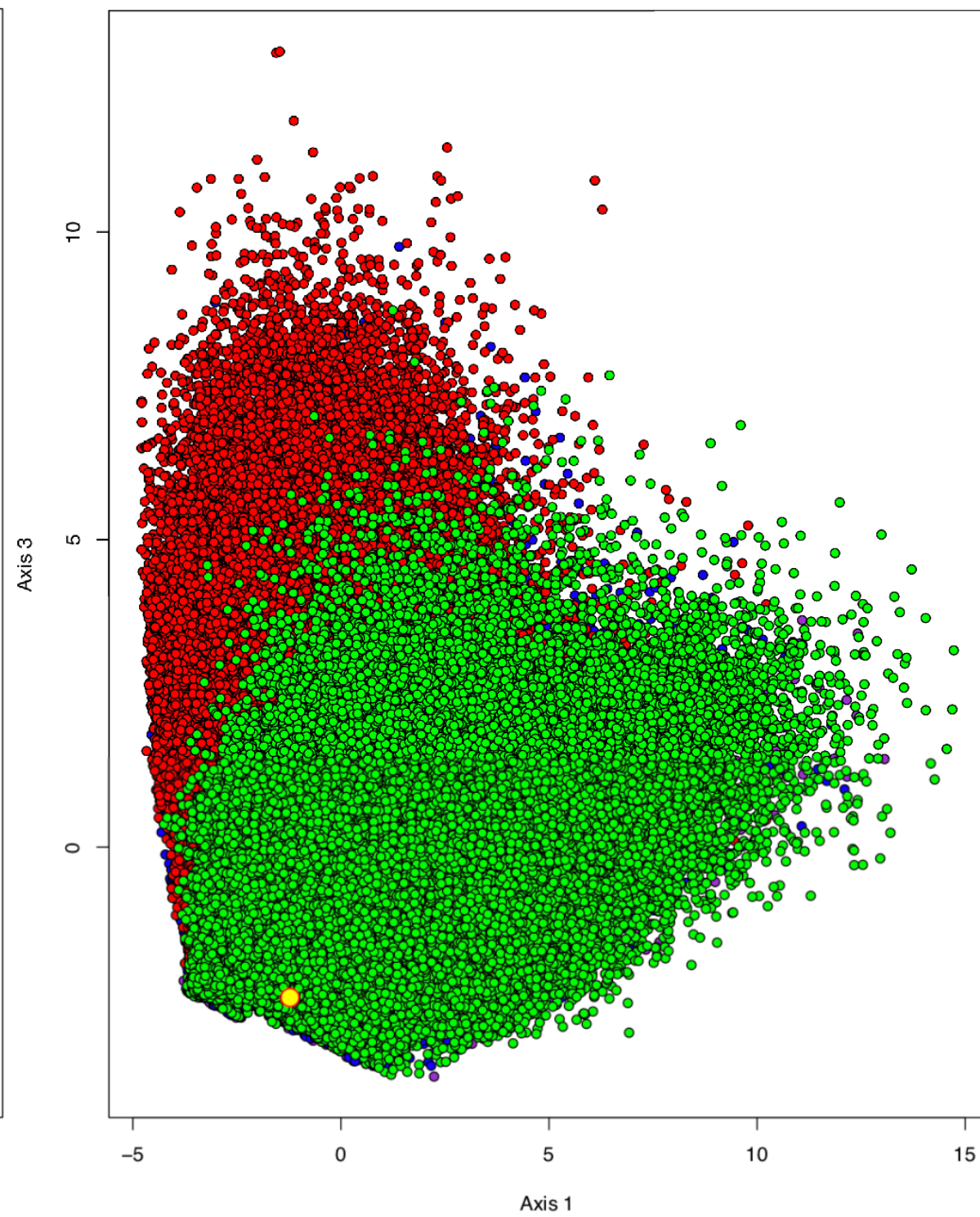

Prior checking

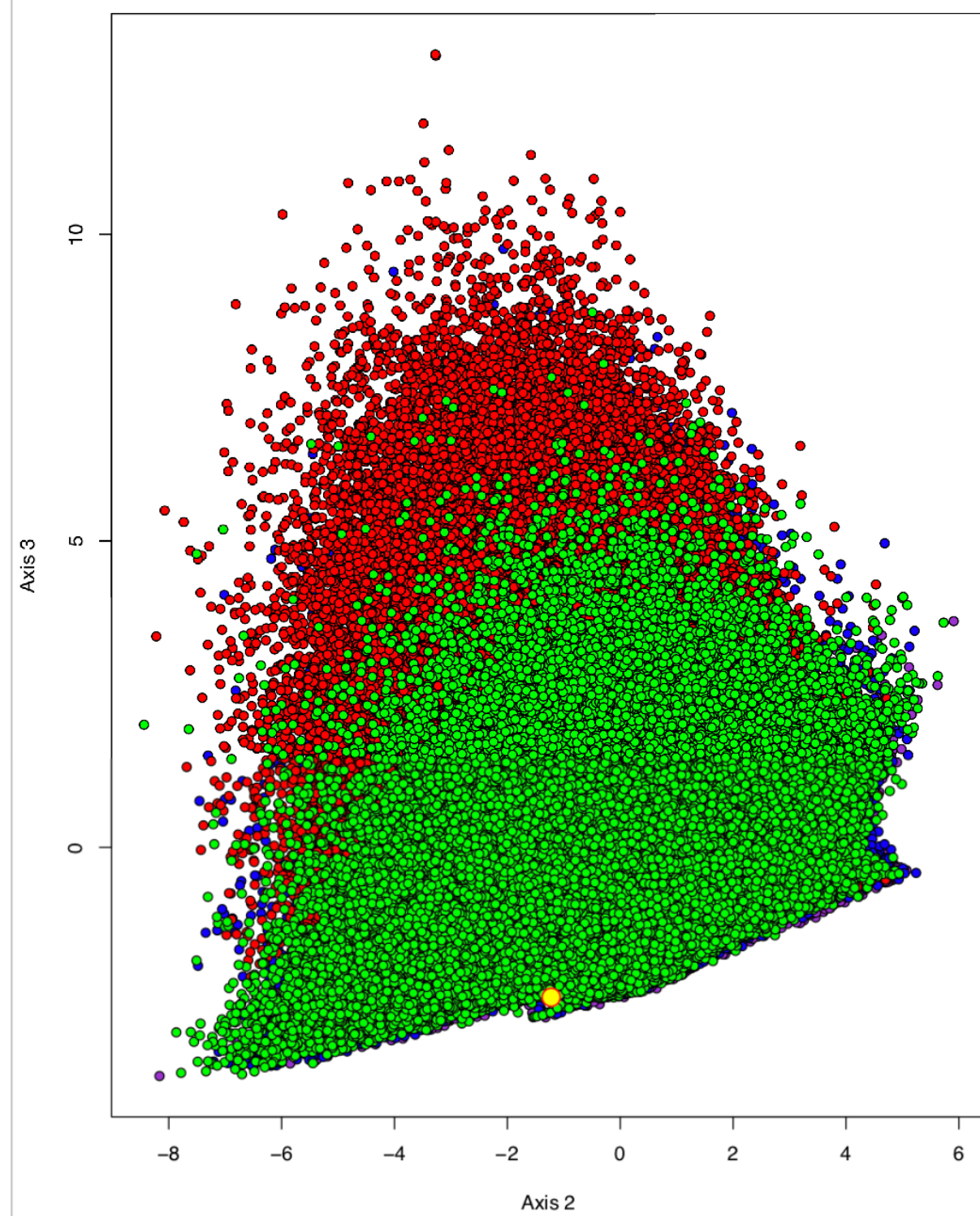

Supplement: Supplementary file 10 [file ECE3-9-12980-s010.pdf]

K\_1

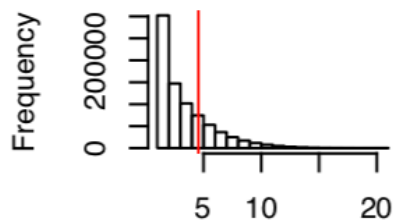

K\_2

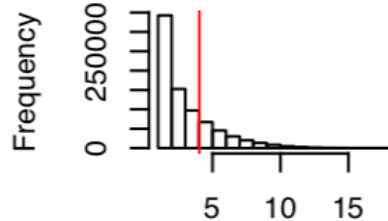

H\_1

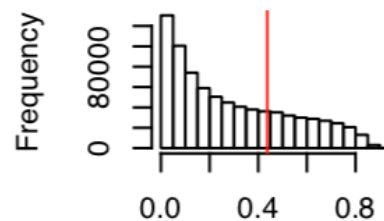

H\_2

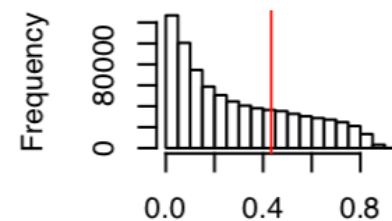

NGW\_1

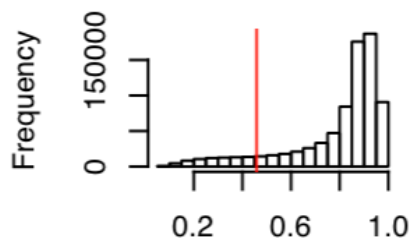

NGW\_2

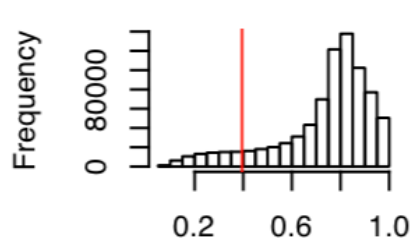

FST\_2\_1

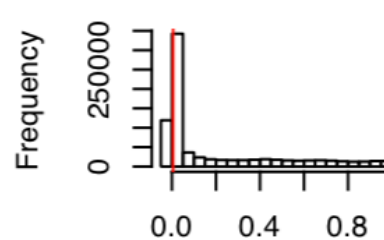

DMUSQ\_2\_1

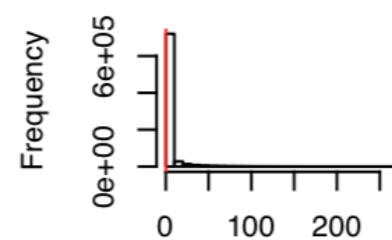

seqK\_1

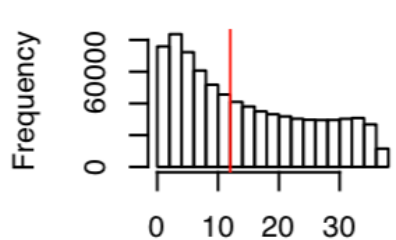

seqK\_2

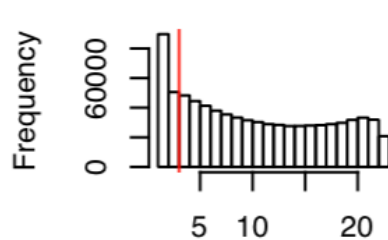

seqH\_1

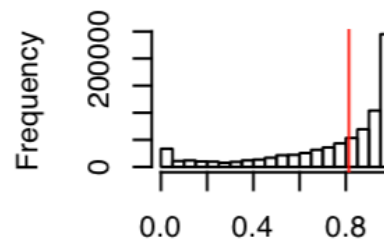

seqH\_2

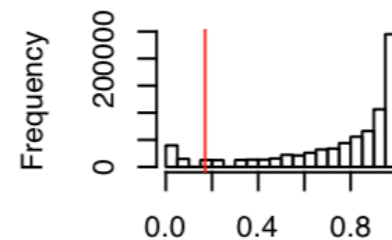

seqD\_1

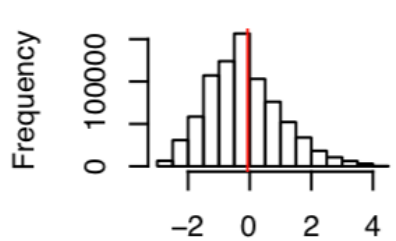

seqD\_2

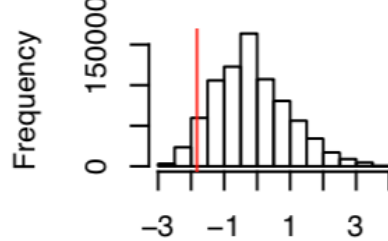

seqFS\_1

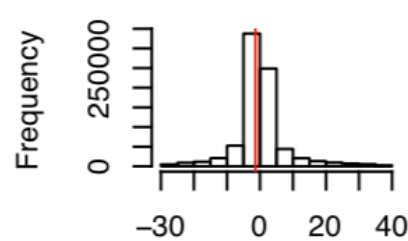

seqFS\_2

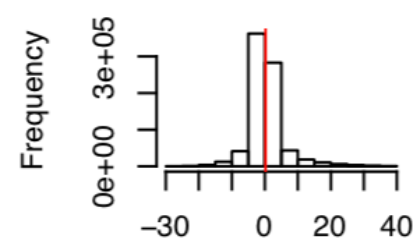

seqPi\_1

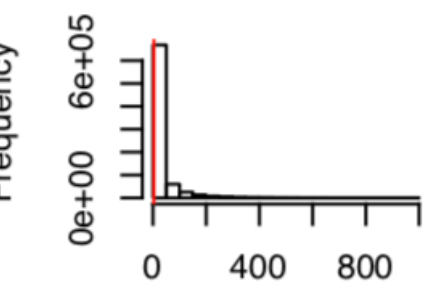

seqPi\_2

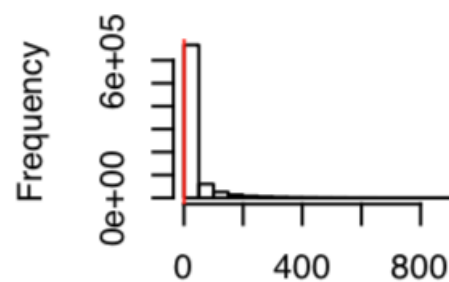

seqFST\_2\_1

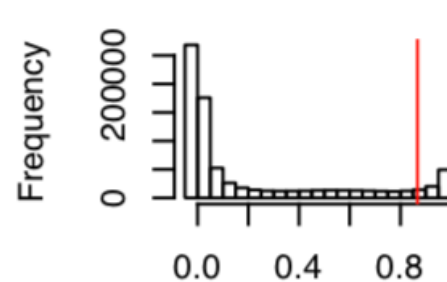

Supplement: Supplementary file 11 [file ECE3-9-12980-s011.pdf]

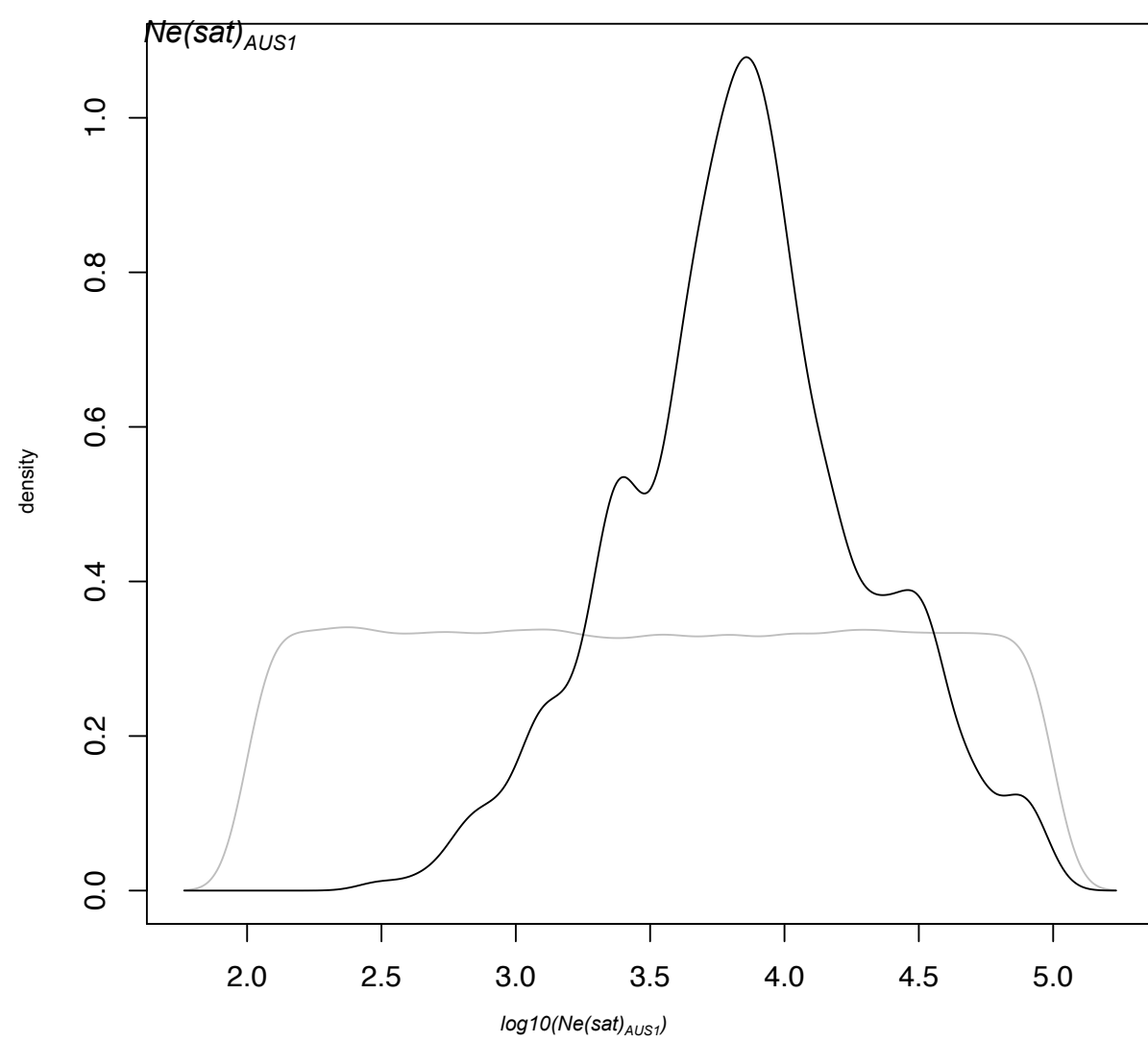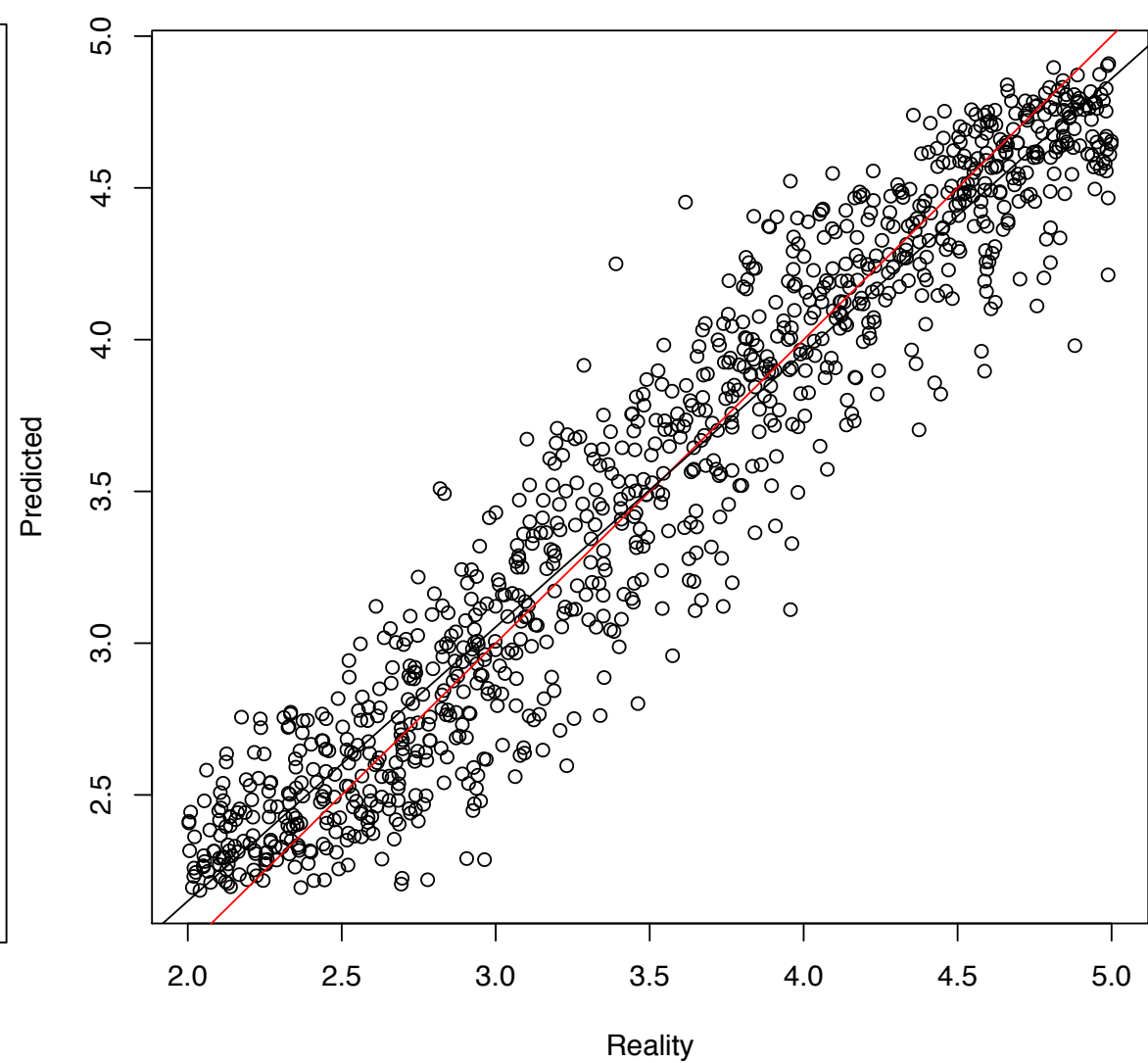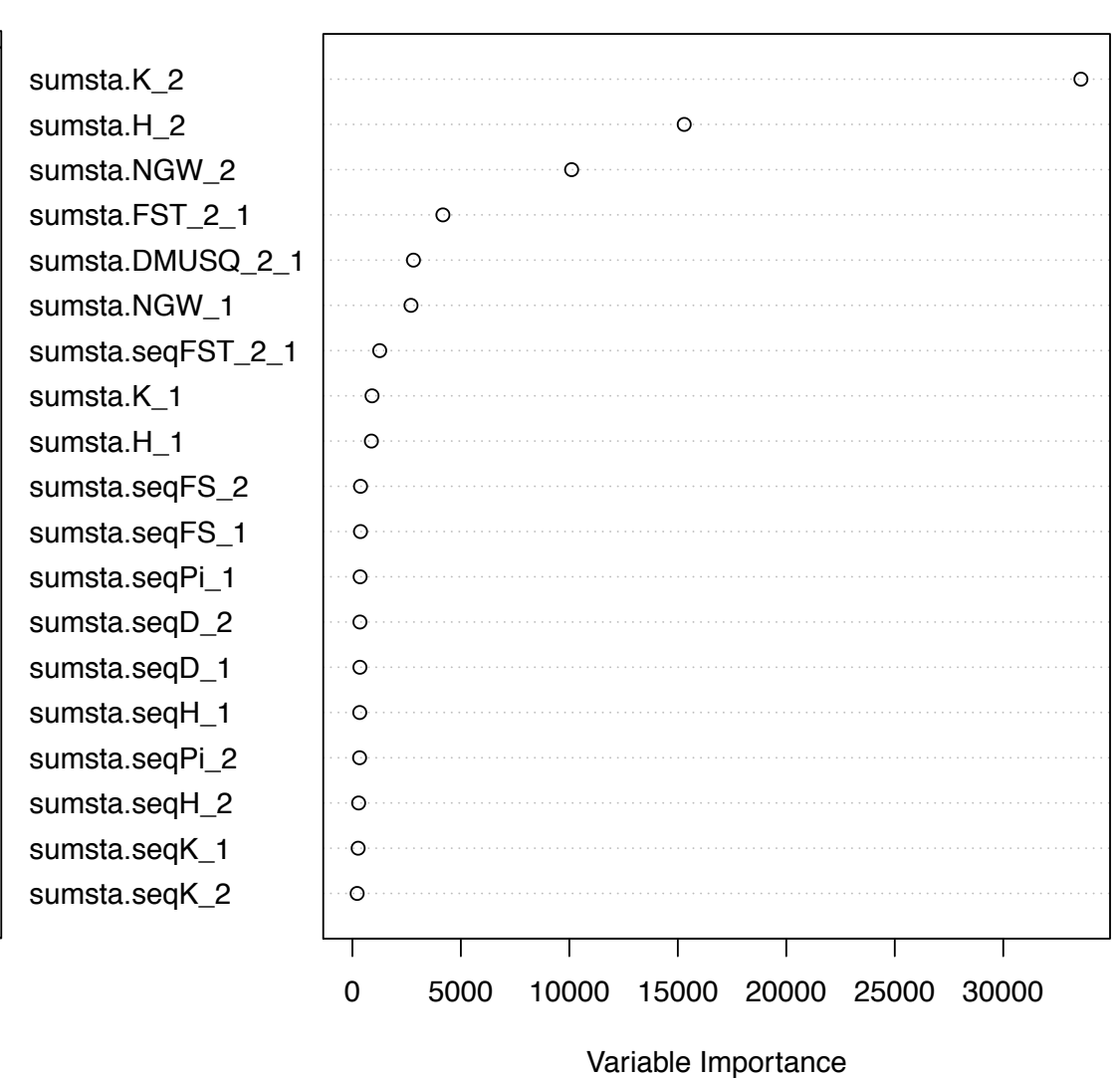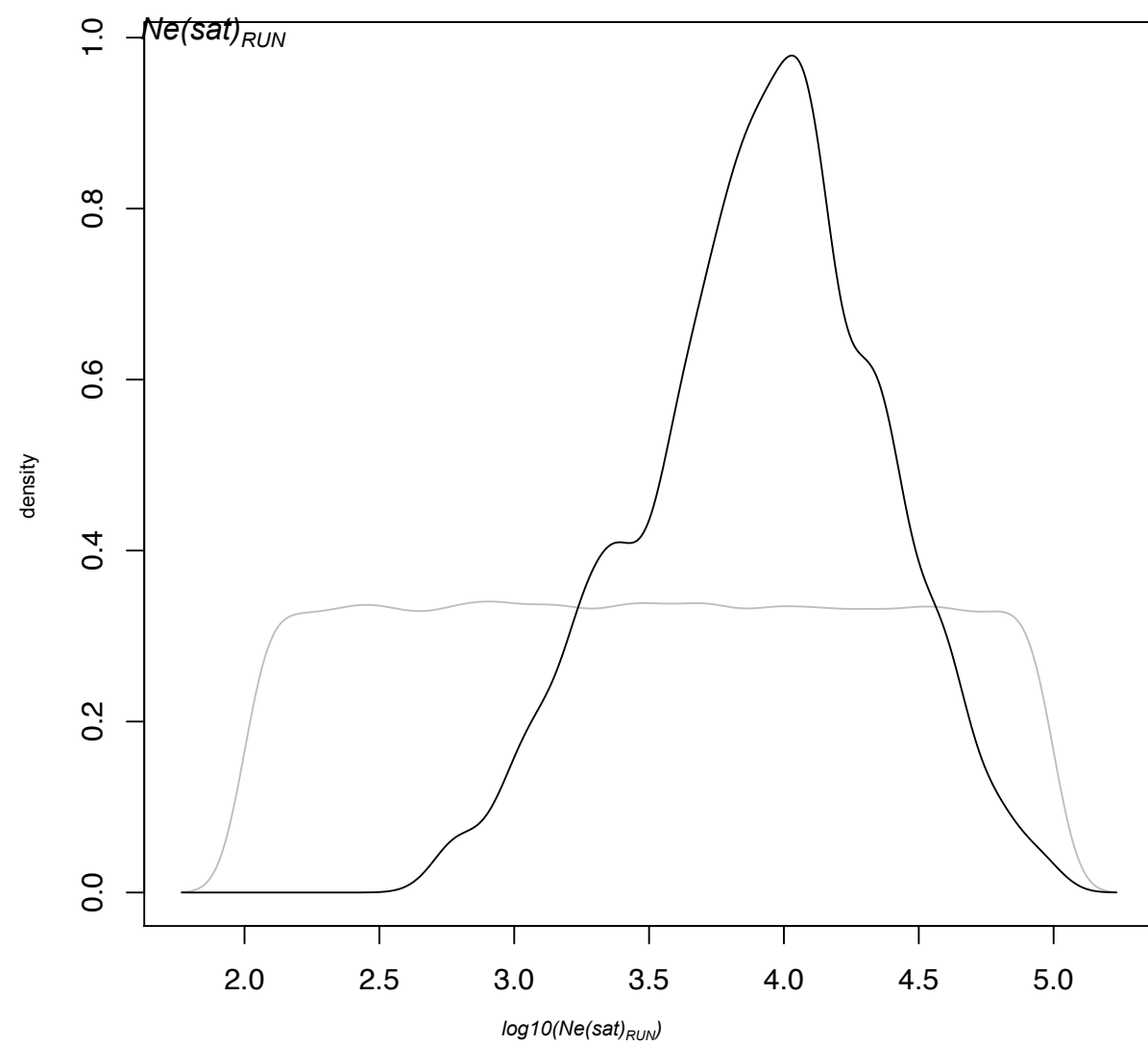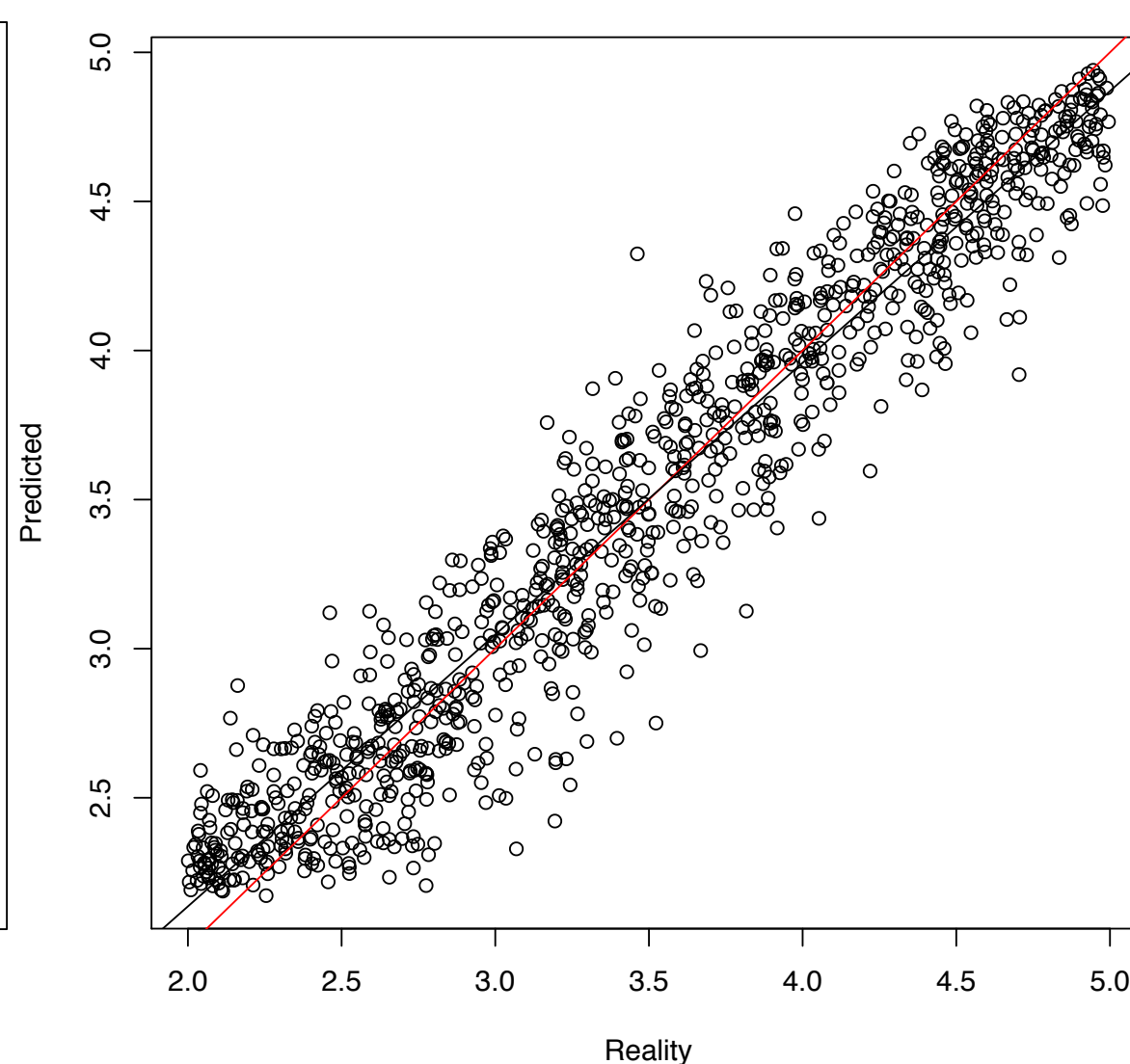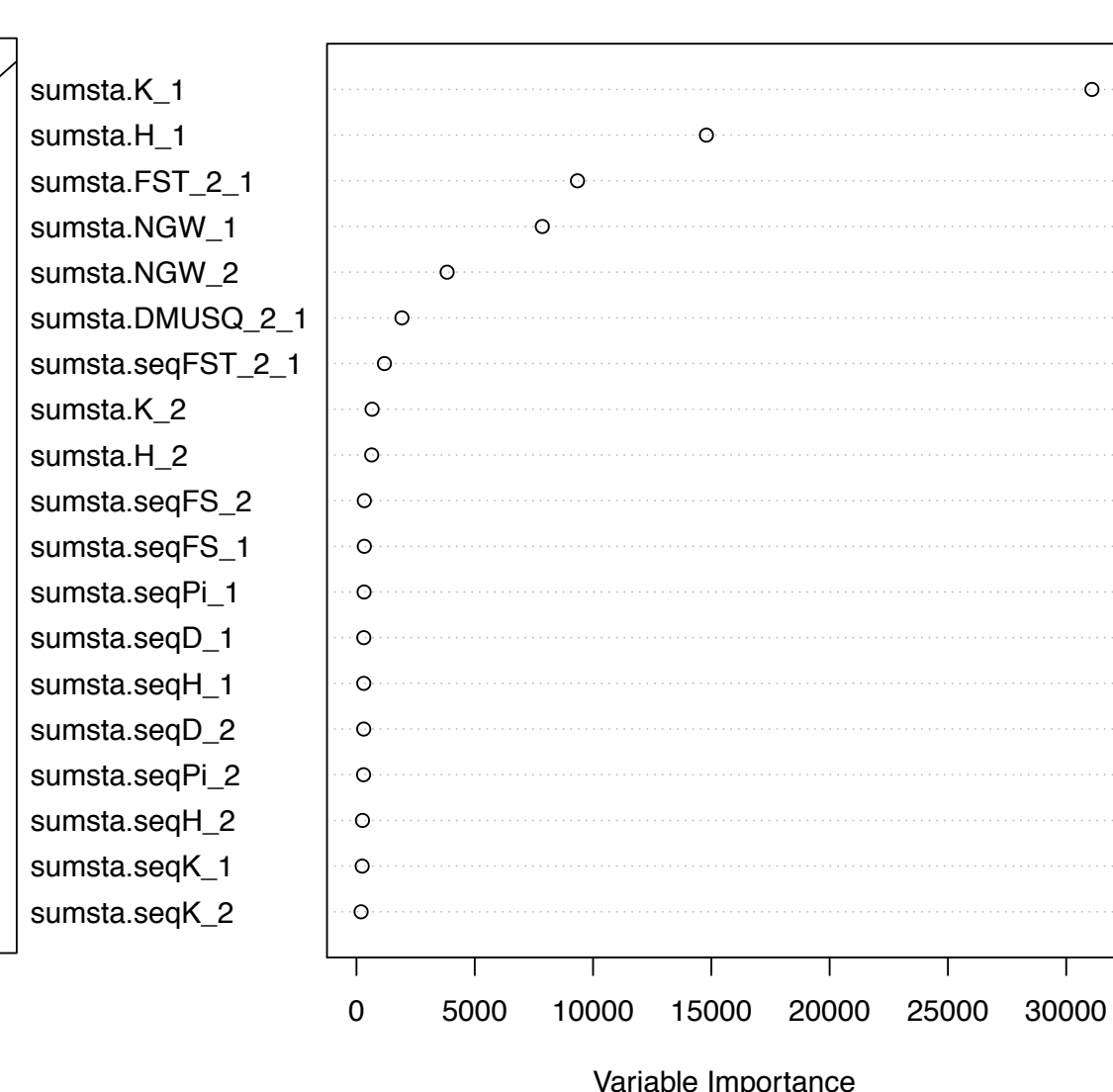

Supplement: Supplementary file 12 [file ECE3-9-12980-s012.pdf]
