# Supplementary material for: Population structure, connectivity, and demographic history of an apex marine predator, the bull shark Carcharhinus leucas
Source: Ecol Evol. 2019 Sep 30;9(23):12980–3000. doi: 10.1002/ece3.5597 (PMC6912899; doi:10.1002/ece3.5597)
Supplement: Supplementary file 13 [file ECE3-9-12980-s013.pdf]

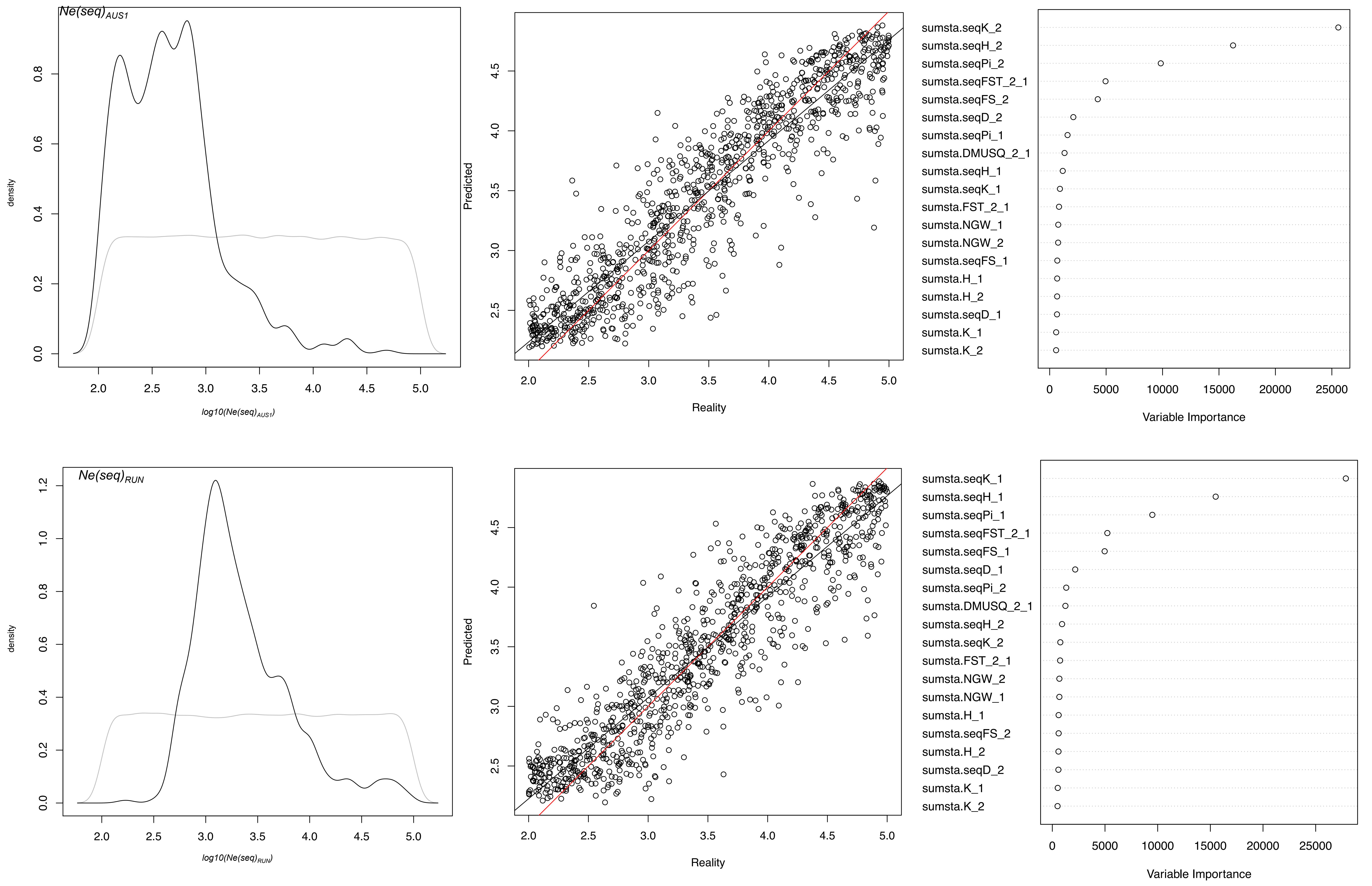

Ne(seq)AUS1
density
log10(Ne(seq)AUS1)
Predicted
Reality
sumsta.seqK_2
sumsta.seqH_2
sumsta.seqPi_2
sumsta.seqFST_2_1
sumsta.seqFS_2
sumsta.seqD_2
sumsta.seqPi_1
sumsta.DMUSQ_2_1
sumsta.seqH_1
sumsta.seqK_1
sumsta.FST_2_1
sumsta.NGW_1
sumsta.NGW_2
sumsta.seqFS_1
sumsta.H_1
sumsta.H_2
sumsta.seqD_1
sumsta.K_1
sumsta.K_2
Variable Importance
Ne(seq)RUN
density
log10(Ne(seq)RUN)
Predicted
Reality
sumsta.seqK_1
sumsta.seqH_1
sumsta.seqPi_1
sumsta.seqFST_2_1
sumsta.seqFS_1
sumsta.seqD_1
sumsta.seqPi_2
sumsta.DMUSQ_2_1
sumsta.seqH_2
sumsta.seqK_2
sumsta.FST_2_1
sumsta.NGW_2
sumsta.NGW_1
sumsta.H_1
sumsta.seqFS_2
sumsta.H_2
sumsta.seqD_2
sumsta.K_1
sumsta.K_2
Variable Importance
