# Supplementary material for: Population structure, connectivity, and demographic history of an apex marine predator, the bull shark Carcharhinus leucas
Source: Ecol Evol. 2019 Sep 30;9(23):12980–3000. doi: 10.1002/ece3.5597 (PMC6912899; doi:10.1002/ece3.5597)
Supplement: Supplementary file 14 [file ECE3-9-12980-s014.docx]

**Appendix A12.** Characteristics of posterior distributions estimated with ABC-RF for all parameters studied. *OOB-MSE,* out-of-bag mean square error; *NMSE,* normalized mean squared error; *NMAE*, normalized mean absolute error; *CI*, 95% confidence interval.

| **Parameter** | **Expectation** | **Median** | **Variance** | **2.5% quantile** | **97.5% quantile** | **OOB-MSE** | **NMSE** | **NMAE** | **Mean relative CI** | **Median relative CI** |
| --- | --- | --- | --- | --- | --- | --- | --- | --- | --- | --- |
| **log10(*Ne(sat)_RUN_*)** | 3.89 (0.01) | 3.90 (0.03) | 0.06 (0.02) | 3.01 (0.00) | 4.73 (0.01) | 0.05 (0.00) | 0.02 | 0.06 | 0.30 | 0.30 |
| **log10(*Ne(sat)_AUS1_*)** | 3.85 (0.00) | 3.86 (0.01) | 0.07 (0.03) | 2.89 (0.03) | 4.8 (0.01) | 0.06 (0.00) | 0.02 | 0.06 | 0.32 | 0.32 |
| **log10(*Ne(sat)_ANC_*)** | 3.71 (0.01) | 3.84 (0.01) | 0.29 (0.09) | 2.12 (0.00) | 4.85 (0.01) | 0.46 (0.01) | 0.16 | 0.17 | 0.73 | 0.67 |
| **log10(*Ne(seq)_RUN_*)** | 3.37 (0.01) | 3.26 (0.02) | 0.17 (0.05) | 2.69 (0.03) | 4.68 (0.02) | 0.10 (0.00) | 0.03 | 0.08 | 0.39 | 0.38 |
| **log10(*Ne(seq)_AUS1_*)** | 2.62 (0.03) | 2.57 (0.03) | 0.16 (0.02) | 2.03 (0.01) | 3.67 (0.04) | 0.11 (0.00) | 0.03 | 0.08 | 0.40 | 0.39 |
| **log10(*Ne(seq)_ANC_*)** | 3.32 (0.08) | 3.34 (0.09) | 0.52 (0.06) | 2.07 (0.01) | 4.74 (0.03) | 0.47 (0.00) | 0.14 | 0.17 | 0.73 | 0.70 |
| ***θsat_RUN_*** | 2.99 (0.03) | 1.75 (0.00) | 3.55 (0.15) | 0.25 (0.17) | 12.18 (0.03) | 0.81 (0.00) | 0.15 | 0.36 | 2.15 | 1.53 |
| ***θsat_AUS1_*** | 3.67 (0.43) | 2.23 (0.42) | 7.31 (1.32) | 0.21 (0.03) | 15.02 (0.45) | 1.10 (0.01) | 0.52 | 0.62 | 3.55 | 1.62 |
| ***θsat_ANC_*** | 2.22 (0.03) | 1.00 (0.01) | 4.80 (0.32) | 0.02 (0.01) | 12.33 (0.09) | 3.94 (0.01) | 25.03 | 12.65 | 61.97 | 5.38 |
| ***θseq_RUN_*** | 0.0020 (0.0074) | 0.0011 (0.0043) | 0.00001 (0.0033) | 0.0004 (0.0008) | 0.0132 (0.0542) | 0.0000 (0.0002) | 0.002 | 0.92 | 5.22 | 2.07 |
| ***θseq_AUS1_*** | 0.0006 (0.0083) | 0.0002 (0.0002) | 0.000005 (0.0019) | 0.0000 (0.0018) | 0.0050 (0.0282) | 0.0000 (0.0001) | 0.002 | 0.96 | 5.72 | 2.54 |
| ***θseq_ANC_*** | 0.0045 (0.0183) | 0.0015 (0.0127) | 0.0001 (0.0036) | 0.0000 (0.0010) | 0.0290 (0.0385) | 0.0001 (0.0001) | 0.10 | 14.04 | 84.02 | 8.65 |
| ***M(sat)_RUN-AUS_*** | 0.101 (0.0070) | 0.103 (0.0152) | 0.004 (0.0005) | 0.005 (0.0037) | 0.197 (0.0005) | 0.003 (0.0000) | 0.17 | 1.96 | 5.00 | 1.92 |
| ***M(sat)_AUS-RUN_*** | 0.103 (0.0149) | 0.103 (0.0190) | 0.004 (0.0048) | 0.006 (0.0225) | 0.196 (0.0002) | 0.003 (0.0001) | 0.19 | 2.21 | 5.44 | 1.89 |
| ***M(seq)_RUN-AUS_*** | 0.101 (0.0054) | 0.103 (0.0161) | 0.004 (0.0027) | 0.004 (0.0017) | 0.196 (0.0025) | 0.003 (0.0000) | 0.14 | 1.74 | 4.59 | 1.84 |
| ***M(seq)_AUS-RUN_*** | 0.100 (0.0036) | 0.100 (0.0004) | 0.004 (0.0024) | 0.004 (0.0142) | 0.196 (0.0035) | 0.003 (0.0001) | 0.20 | 2.34 | 5.71 | 1.88 |
