# Supplementary material for: Population structure, connectivity, and demographic history of an apex marine predator, the bull shark Carcharhinus leucas
Source: Ecol Evol. 2019 Sep 30;9(23):12980–3000. doi: 10.1002/ece3.5597 (PMC6912899; doi:10.1002/ece3.5597)
Supplement: Supplementary file 15 [file ECE3-9-12980-s015.docx]

# Appendices

**Table of contents**

**Appendix A1.** Panels used to multiplex loci post-PCR and allelic ranges (in base pairs, without M13-tail). 47

**Appendix A2.** Priors and summary statistics used for the ABC-RF analyses. 48

**Appendix A3.** Summary statistics for each sampling location for the three mitochondrial markers used, the control region *CR* (923 bp), *nd4* (672 pb), and *cytb* (921 bp). 49

**Appendix A4.** Posterior trace files of the BEAST analyses calibrated (a) using the divergence date between *Carcharhinus* and *Sphyrna* genera, 38 Mya; (b) using the formation of the Isthmus of Panama 3.1-3.5 Mya. 50

**Appendix A5.** Geographic distribution of mitochondrial concatenated sequence *CR-nd4-cytb* haplotypes found in bull sharks from the Western Indian Ocean (WIO), the Western Pacific (WP), and the Western Atlantic (WA). 51

**Appendix A6.** Average probability of membership (*y*-axis) of *Carcharhinus leucas* individuals (*N* = 357, *x-*axis) to putative clusters assuming correlated allele frequencies and admixture as performed by Structure. 52

**Appendix A7.** Analyses of molecular variance performed for 25 microsatellite loci and three mitochondrial loci concatenated (*CR-nd4-cytb*). 54

**Appendix A8.** *Carcharhinus leucas* demography statistics (Tajima’s *D* and Fu’s *F_S_*) for the concatenated mitochondrial sequence *CR-nd4-cytb*. 55

**Appendix A9.** Principal component analyses of summary statistics simulated under the four demographic scenarios for the bull shark populations from the Western Indian Ocean (represented by RUN) and from the Western Pacific (represented by AUS1). 56

**Appendix A10.** Distribution of the summary statistics for the simulated datasets. ………...57

**Appendix A11.** Effective population size estimates under the best scenario for bull shark populations from the Western Indian Ocean (represented by RUN) and from the Western Pacific (represented by AUS1). 58

**Appendix A12.** Characteristics of posterior distributions estimated with ABC-RF for all parameters studied. 60

**Appendix A1.** Panels used to multiplex loci post-PCR and allelic ranges (in base pairs, without M13 tail).

**Appendix A2.** Priors and summary statistics used for the ABC-RF analyses.

**Table A2.1.** Priors for the demographic parameters

**Table A2.2.** Summary statistics of the observed datasets estimated for the ABC analyses. For microsatellites: *K*¸ mean number of alleles over loci; *H*, mean of Nei’s gene diversity; *MGW*, mean over loci of the modified Garza–Williamson index; *F_ST_*, pairwise microsatellite *F_ST_*; *DMUSQR*, mean delta mu-square (square difference in mean microsatellite allele length between pairs of populations). For the mitochondrial marker: *seqK*¸ mean number of alleles over loci; *seqH*, mean of Nei’s gene diversity; *Pi*, mean number of pairwise differences; *D*, Tajima’s *D*; *Fs*, Fu’s *Fs*; *seqF_ST_*, pairwise *F_ST_*.

**Appendix A3.** Summary statistics for each sampling location for the three mitochondrial markers used, the control region *CR* (923 bp), *nd4* (672 pb), and *cytb* (921 bp). ZAN, Zanzibar; SEY, Seychelles; MOZ, Mozambique; SAF, South Africa; MAD, Madagascar; RUN, Reunion Island; ROD, Rodrigues Island; AUS1, Clarence River, Australia; AUS2, Sydney Harbour, Australia; NCA, New Caledonia; FLO, Florida. *N_s_*, number of individuals sequenced; *H*, number of haplotypes, *h*, haplotype diversity; *S*, number of polymorphic sites; *π*, nucleotide diversity. In parentheses, are indicated standard errors.

**Appendix A4.** Posterior trace files of the BEAST analyses calibrated (a) using the divergence date between *Carcharhinus* and *Sphyrna* genera, 38 Mya; (b) using the formation of the Isthmus of Panama 3.1-3.5 Mya. Concatenated runs of the five analyses performed for each calibration are shown, as well as the effective sample sizes (ESS) of the posterior distribution and of the mean rate of substitution.

**Table A4.1.** Effective sample sizes (ESS) of the posterior distribution and of the mean rate of substitution.

**Figure A4.1.** Concatenated runs of the five analyses performed for each calibration, (a) using the divergence date between *Carcharhinus* and *Sphyrna* genera, 38 Mya; (b) using the formation of the Isthmus of Panama 3.1-3.5 Mya.

A**ppendix A5.** Geographic distribution of mitochondrial concatenated sequence *CR-nd4-cytb* haplotypes found in bull sharks from the Western Indian Ocean (WIO), the Western Pacific (WP), and the Western Atlantic (WA). ZAN, Zanzibar; SEY, Seychelles; MOZ, Mozambique; SAF, South Africa; MAD, Madagascar; RUN, Reunion Island; ROD, Rodrigues Island; AUS1, Clarence River, Australia; AUS2, Sydney Harbour, Australia; NCA, New Caledonia; FLO, Florida.

**Appendix A6.** Average probability of membership (*y*-axis) of *Carcharhinus leucas* individuals (*N* = 357, *x-*axis) to putative clusters assuming correlated allele frequencies and admixture as performed by Structure, *K* varying from two to five (10 runs for each *K*). (a) 25 microsatellites and no *a priori* sampling location information, (b) 25 microsatellites and the LOCPRIOR model, (c) 25 microsatellites and the concatenated mitochondrial sequence *CR-nd4-cytb*, no *a priori* sampling location information, and (d) 25 microsatellites and the concatenated mitochondrial sequence *CR-nd4-cytb*, with the LOCPRIOR model. Only major modes are presented, and the averaged log likelihood of observing the data across the 10 runs ± standard deviation is indicated for the best *K* for each analysis (*LnLk*). ZAN, Zanzibar; SEY, Seychelles; MOZ, Mozambique; SAF, South Africa; MAD, Madagascar; RUN, Reunion Island; ROD, Rodrigues Island; AUS1, Clarence River, Australia; AUS2, Sydney Harbour, Australia; NCA, New Caledonia; FLO, Florida.

**Appendix A7.** Analyses of molecular variance performed for 25 microsatellite loci and three mitochondrial loci concatenated (*CR-nd4-cytb*). **: *P* < 0.01; ***: *P* < 0.001.

**Appendix A8.** *Carcharhinus leucas* demography statistics (Tajima’s *D* and Fu’s *F_S_*) for the concatenated mitochondrial sequence *CR-nd4-cytb* (ZAN, Zanzibar; SEY, Seychelles; MOZ, Mozambique; SAF, South Africa; MAD, Madagascar; RUN, Reunion Island; ROD, Rodrigues Island; AUS1, Clarence River, Australia; AUS2, Sydney Harbour, Australia; NCA, New Caledonia; FLO, Florida). All values were not significantly different from zero (*P*> 0.05).

**Appendix A9.** Principal component analyses of summary statistics simulated under the four demographic scenarios for the bull shark populations from the Western Indian Ocean (represented by RUN) and from the Western Pacific (represented by AUS1). In purple are simulations of Scenario 1; in blue, simulations of Scenario 2; in red, simulations of Scenario 3; and in green, simulations of Scenario 4. Percentage of variance explained by each axis, Axis 1: 20.31%; Axis 2: 17.55%; and Axis 3: 16.13%. The observed dataset is represented by the yellow point.

**Appendix A10.** Distribution of the summary statistics for the simulated datasets. In red is represented the summary statistics value of the observed dataset. For microsatellites: *K*¸ mean number of alleles over loci; *H*, mean of Nei’s gene diversity; *NGW*, mean over loci of the modified Garza–Williamson index; *F_ST_*, pairwise microsatellite *F_ST_*; *DMUSQR*, mean delta mu-square (square difference in mean microsatellite allele length between pairs of populations). For the mitochondrial marker: *seqK*¸ mean number of alleles over loci; *seqH*, mean of Nei’s gene diversity; *Pi*, mean number of pairwise differences; *D*, Tajima’s *D*; *Fs*, Fu’s *Fs*; *seqF_ST_*, pairwise *F_ST_*. For each summary statistic, 1 refers to RUN and 2 to AUS1.

**Appendix A11.** Effective population size estimates under the best scenario for bull shark contemporary populations from the Western Indian Ocean (represented by RUN) and from the Western Pacific (represented by AUS1) with (a) microsatellite (*Ne(sat)*) and (b) mtDNA (*Ne(seq)*). Left column: prior (dotted line) and posterior (plain line) distributions of the effective population sizes. Central column: correlation between effective population sizes simulated in the 1,000 pseudo-observed datasets and their corresponding estimates using the fitted model. Right column: contribution of each summary statistics to parameter estimation.

**Appendix A12.** Characteristics of posterior distributions estimated with ABC-RF for all parameters studied. *OOB-MSE,* out-of-bag mean square error; *NMSE,* normalized mean squared error; *NMAE*, normalized mean absolute error; *CI*, 95% confidence interval.
